# Discovery of Genomic Targets and Therapeutic Candidates for Liver Cancer Using Single-Cell RNA Sequencing and Molecular Docking

**DOI:** 10.3390/biology14040431

**Published:** 2025-04-17

**Authors:** Biplab Biswas, Masahiro Sugimoto, Md. Aminul Hoque

**Affiliations:** 1 Department of Statistics, Faculty of Science, Gopalganj Science & Technology University, Gopalganj 8100, Bangladesh; bbiswas@isrt.ac.bd; 2 Department of Statistics, Faculty of Science, University of Rajshahi, Rajshahi 6205, Bangladesh; 3 Institute for Advanced Biosciences, Keio University, Tsuruoka 997-0052, Japan; msugi@sfc.keio.ac.jp; 4 Institute of Medical Science, Tokyo Medical University, Tokyo 160-8402, Japan

**Keywords:** liver cancer, scRNA-seq, genomic, survival analysis, drug suggestion

## Abstract

Liver cancer is one of the most common malignancies and the second leading cause of cancer-related deaths worldwide, particularly in developing countries, where it poses a significant financial burden. Early detection and timely treatment remain challenging due to the complex mechanisms underlying the initiation and progression of liver cancer. This study aims to uncover key genomic features, analyze their functional roles, and propose potential therapeutic drugs identified through molecular docking, utilizing single-cell RNA sequencing (scRNA-seq) data from liver cancer studies. We applied two advanced hybrid methods known for their robust identification of differentially expressed genes (DEGs) regardless of sample size, along with four top-performing individual methods. These approaches were used to analyze four scRNA-seq datasets, leading to the identification of essential DEGs. Through a protein−protein-interaction (PPI) network, we identified 25 hub-of-hub genes (hHubGs) and 20 additional hHubGs from two naturally occurring gene clusters, ultimately validating a total of 36 hHubGs. Functional, pathway, and survival analyses revealed that these hHubGs are strongly linked to liver cancer. Based on molecular docking and binding-affinity scores with 36 receptor proteins, we proposed 10 potential therapeutic drugs, which we selected from a pool of 300 cancer meta-drugs. The choice of these drugs was further validated using 14 top-ranked published receptor proteins from a set of 42. The proposed candidates include Adozelesin, Tivozanib, NVP-BHG712, Nilotinib, Entrectinib, Irinotecan, Ponatinib, and YM201636. This study provides critical insights into the genomic landscape of liver cancer and identifies promising therapeutic candidates, serving as a valuable resource for advancing liver cancer research and treatment strategies.

## Simple Summary

This study aimed to identify potential hub genes and perform functional analysis using these genes, as well as to suggest drugs identified through molecular docking based on analysis of scRNA-seq data from liver cancer studies. To find differentially expressed genes using scRNA-seq data, we used two recently developed hybrid methods and four top-performing individual methods. Functional and pathway analyses indicated that the functions/pathways identified by hHubGs are closely associated with liver cancer. Finally, we recommend Adozelesin, Tivozanib, NVP-BHG712, Nilotinib, Entrectinib, Irinotecan, Potatinib, and YM201636 as candidate drugs for liver cancer.

## 1. Introduction

Liver cancer originates in the liver as a malignant tumor and has one of the highest incidence rates among cancers globally [1]. According to the American Cancer Society, liver cancer accounts for over 700,000 deaths annually worldwide, making it the second leading cause of cancer-related mortality. The two primary types of liver cancer are hepatocellular carcinoma (HCC), the most prevalent form [2], and cholangiocarcinoma. Liver cancer incidence and fatality rates are notably higher in developing nations [3]. Major risk factors for liver cancer include infection with hepatitis B virus or hepatitis C virus, hepatic cirrhosis, and alcoholic liver disease [4,5,6]. Evidence from various studies suggests that the molecular pathogenesis of liver cancer, including HCC, is closely linked to genetic predispositions and environmental influences, such as oncogene activation, gene mutations, and the inactivation of tumor suppressor genes [7,8]. Despite these findings, the mechanisms underlying the progression and development of liver cancer remain incompletely understood. Moreover, one of the leading causes of liver cancer mortality is its asymptomatic nature during the early stages, which makes early diagnosis challenging. Consequently, the disease is difficult to treat, has a poor prognosis, and is associated with a high recurrence rate [2]. Therefore, identifying precise biomarkers and accurately predicting liver cancer progression holds significant potential to mitigate its severity and improve clinical outcomes.

The identification of key therapeutic targets offers a viable approach to effectively addressing diseases. Numerous studies have attempted to identify critical genes associated with liver cancer. To identify the key genes and pathways involved in hepatocellular carcinoma, Wu et al. conducted a bioinformatics analysis using microarray data and conventional methods [2]. Li et al. used the classical *t*-test and microarray data to identify the key genes involved in hepatocellular carcinoma [9]. Some other studies have also conducted analyses based on conservative microarray data or bulk RNA-seq data to identify genes potentially involved in liver cancer [10,11,12]. However, the findings have often lacked consistency, and analyses based on single-cell RNA sequencing (scRNA-seq) data have not yet been conducted. However, single-cell RNA-seq analysis has some innovative features compared to microarray or bulk RNA-seq data like multimodality, excess zeros, and cellular heterogeneity, which are important for the accurate detection of differentially expressed genes [13,14,15]. Notably, scRNA-seq data analysis offers a promising avenue for the study of liver cancer due to its unique and complex characteristics, such as excess zeros, multimodality, high dispersion, and cellular heterogeneity. Thus, a comprehensive study leveraging scRNA-seq data is necessary if we are to accurately identify the biomarkers underlying liver cancer and select the most effective candidate drugs based on these biomarkers. However, the development of novel drugs for liver cancer remains a challenging, time-intensive, and costly endeavor. To address this, it is essential to explore target proteins, known as receptors, that play a role in liver cancer pathogenesis and identify small molecules (drug agents) capable of mitigating the disease through interaction with these receptors. Consequently, identifying an appropriate drug from the pool of existing drugs for a specific disease could significantly reduce time and costs compared to developing entirely new drugs.

## 2. Methods and Materials

### 2.1. Data Description

The datasets utilized in this study include GSE146409 [16], GSE202069 [17], GSE189935, and GSE98638 [18]. The GSE98638 dataset comprises CD8+ T cells from adjacent normal liver tissues (NTC) and CD8+ T cells from liver tumor tissues (TTC). To ensure data quality, we curated the dataset so that each gene contained at least 10% non-zero observations. Initially, this dataset included 23,387 genes and 1189 samples (412 NTC vs. 777 TTC). After curation, the dataset retained 9288 genes. Similarly, the other datasets were curated to ensure that each gene contained at least 10% non-zero observations. The case vs. control groups in these datasets were as follows:

GSE146409: Non-malignant liver tumor (12) vs. malignant liver tumor (63).

GSE202069: Non-tumor (25) vs. tumor (41).

GSE189935: Adjacent normal tissues (987) vs. tumor tissues (2199).

Detailed descriptions of these datasets are provided in Table 1.

### 2.2. Hybrid Model

#### 2.2.1. scDEA

scDEA [19] is an ensemble learning-based differential expression analysis method designed to produce more accurate and stable results than 12 individual differential expression (DE) analysis methods. Lancaster’s combination method integrates the *p*-values generated by these individual methods.

The scDEA method involves three primary steps:

1. Normalization: Normalize the scRNA-seq count data matrix according to the requirements of the 12 individual methods by using the count-per-million (CPM) method.

2. *p*-value Generation: Compute the *p*-values for each gene using all 12 methods.

3. Integration: Combine the *p*-values using Lancaster’s combination method by adjusting weights based on rank correlation coefficients to produce a single, combined *p*-value for each gene.

This method demonstrates robustness and stability in identifying DEGs, particularly in scenarios with limited sample sizes, outperforming individual methods regarding reliability and accuracy. We used a trim parameter of 0.2 in scDEA as a default.

#### 2.2.2. scHD4E

scHD4E [15] is a recently developed hybrid model (2024) that outperforms the existing hybrid model (scDEA) and the top-ranked four individual methods (ROSeq, TPMM, Seurat, Limma (voom)), as well as the 12 individual methods. The four top-ranked methods were selected through a rigorous evaluation process from a pool of 44 differential expression (DE) analysis methods for single-cell RNA-sequencing data [15].

Using Lancaster’s combination method, this model integrates the *p*-values generated exclusively by these four methods. A comprehensive experiment to assess its performance was conducted across five real-world scRNA-seq datasets. It evaluated the method from multiple perspectives, including sample-size effects, batch effects, control of type I error, runtime, and gene-enrichment analysis. scHD4E demonstrated exceptional performance in all these aspects, surpassing the other methods in accuracy, robustness, and efficiency. We applied scHD4E to the count data matrix using a trim parameter of 0.2.

### 2.3. Individual Methods of Differential Expression Analysis 

#### 2.3.1. TPMM

A logistic regression model and linear regression model were used in a two-part mixed model (TPMM) for scRNA-seq data analysis, where logistic regression was used to model the zero proportions and log-transformed non-zero expression values were modeled by linear regression. To implement this mixed model, gene-expression values for single-cell samples in subjects should be normalized. Covariates such as biological condition or clustering effects were incorporated and analyzed using the model. We clustered the cells in our study using the k-means clustering technique, and the “optimcluster” function from the SwarnSeq R package was used to determine the number of clusters. The zlm function contains different methods, but we used “bayesglm”. The relevant R functions are available from the following github link: https://github.com/shilab2017/two_part_mixed_model (accessed on 5 November 2024). The parameters of the model were estimated using marginal maximum likelihood estimation, and differentially expressed genes were identified by testing the fixed effects across biological condition.

#### 2.3.2. ROSeq

The ROSeq model was developed based on discrete generalized beta distribution (DGBD) and models expression ranks as robust representatives if transcript abundance. By applying the DGBD rank-order distribution and ordering the ranks with bins based on bin-wise cellular frequencies, the authors investigated the advantages of discretizing bins of gene expression vectors. The model has two shape parameters, estimated by MLEs for each gene, and genes were filtered using $R^{2}$ as a qualification criterion. To identify the DE genes, the Wald-type test was applied. The ROSeq R package is available from Github at the following link: https://github.com/krishan57gupta/ROSeq (accessed on 6 November 2024).

#### 2.3.3. Limma (Voom)

Limma (a linear model) was developed primarily for analyzing microarray data and conducting DE analysis. To analyze high-dimensional data like RNA-seq, it aggregates a set of statistical principles. Differential gene expression is analyzed by fitting a linear model to each row of the expression matrix, capturing various levels of variability between samples, accounting for complex experimental designs, and borrowing strength from gene-wise models. In our study, the following procedures were used: (i) to design a suitable biological condition for limma, the “model.matrix” R function was used; (ii) using “calcNormFactors” and “voom”, the count expression matrix was normalized by a factor derived from library size; (iii) the “lmFit” R function was used to fit the multiple linear regression by applying generalized least squares; (iv) to identify the DE genes for the multiple linear regression model, the empirical Bayes statistic was used by implementing the “ebayes” function. The Limma R packages included all the above functions.

#### 2.3.4. Seurat

Seurat was developed for quality control, exploration, and analysis of scRNA-seq data by multiple DE analysis methods. In our study, we used SeuratBimon, a model with two states. The first state represents cases where the gene-expression level is not zero and the gene is expressed in a single cell. The second state indicates that the gene-expression level is zero, meaning that the gene is not expressed. In this model, Bernoulli distributions are used to model the zero elements and log-normal distributions are used to model the nonzero elements. To detect the DE genes, a combined likelihood ratio test was performed. The Seurat R package is available from the following link: https://github.com/satijalab/seurat (accessed on 10 November 2024).

### 2.4. Overview of the Analysis Process

We utilized four scRNA-seq datasets from liver cancer samples with the following accession numbers: A = GSE98638, B = GSE146409, C = GSE202069, and D = GSE189935. Two of these datasets—GSE146409 (case = 12 vs. control = 63) and GSE202069 (case = 25 vs. control = 41)—have smaller sample sizes. We applied both the hybrid models and the top-performing individual methods to all four datasets to detect differentially expressed genes (DEGs) with adjusted *p*-values ≤ 0.05. We then identified the intersecting genes between scHD4E and scDEA for all datasets (scDEA ∩ scHD4E). Additionally, we identified the common genes identified by six methods, including the hybrid and individual top four methods (scDEA ∩ scHD4E ∩ Seurat ∩ Limma ∩ TPMM ∩ ROSeq).

Given that we used four datasets, it was possible to identify only a limited number of intersecting genes if we considered the intersection across all four datasets. To address this, we instead focused on the common genes across three datasets for each case, using four combinations of datasets, as follows:

A ∩ B ∩ C

A ∩ B ∩ D

B ∩ D ∩ C

A ∩ D ∩ C

**Figure 1:**
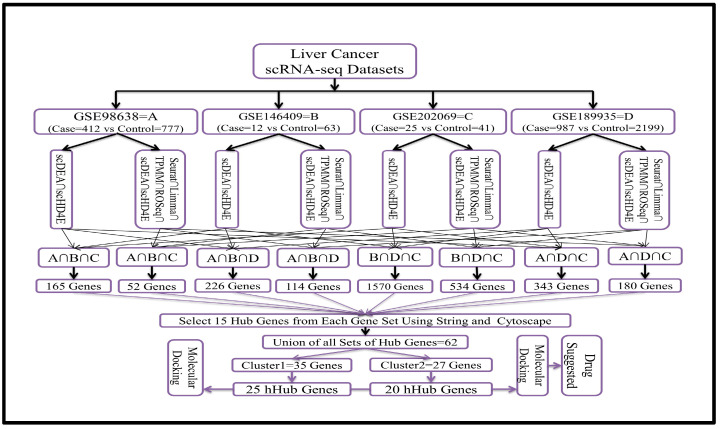
Flow diagram of the entire analysis procedure used to identify drugs for suggestion.

Similarly, for the six methods, we took the common genes from the same four combinations of datasets. For each gene set, we selected 15 hub genes using the STRING database and Cytoscape software (version 3.10.1). This approach yielded 62 genes from the union of all hub gene sets. These genes were divided into two clusters using STRING and Cytoscape. The first cluster contained 35 genes, and the second cluster contained 27 genes. We then selected 25 and 20 hub-of-hub genes (hHubGs) using the cytoHubba plugin for Cytoscape. The entire procedure is illustrated in Figure 1.

### 2.5. Construction of PPI Network for DEGs

To identify hub genes (HubGs), constructing a protein−protein-interaction (PPI) network is essential. Understanding the interactions between proteins in larger protein groups is crucial for functional analysis [20]. In a PPI network, vertices represent proteins and the interactions between proteins are depicted as edges. The PPI network was constructed using the online STRING database [21]. Proteins interacting with many other proteins with a high degree of connectivity are considered to play a central regulatory role and are termed regulatory hubs [22]. For the STRING PPI analysis, we set the confidence score threshold to ≥0.40.

The app cytoHubba in Cytoscape was employed to identify the hub genes by ranking the nodes in the PPI network [23,24]. We selected the 15 top-ranked genes for each set using cytoHubba from the PPI network of intersecting common genes.

### 2.6. Gene Ontology and Pathway Enrichment Analysis

To perform gene ontology (GO) and pathway-enrichment analysis of hHub-DEGs, we utilized four online databases: DAVID [25], STRING [18], Enrichr [26], and WebGestalt [27]. This analysis provided valuable insights into the functional roles of the hub-DEGs. The top hHub-DEGs identified in our study were used to search these databases to analyze the associated biochemistry GO terms, which included Biological Process, Cellular Component, and Molecular Function [28], as well as pathways potentially linked to the progression and development of liver cancer.

We selected the top-ranked biological processes, molecular functions, and cellular components commonly identified by at least two of the four online analysis databases. In most cases, the *p*-values were extremely small (*p* < 0.00001), indicating high statistical significance. Similarly, we conducted pathway enrichment analysis to explore pathways associated with the prognosis and development of liver cancer. We considered widely used pathway databases such as KEGG, WikiPathways, and Reactome, which are commonly employed to detect significantly enriched terms and pathways related to the hHubGs.

### 2.7. Validation of Differential Expression Analysis of hHub Genes

The hub genes identified in our study exhibit differential expression based on the differential-expression analysis performed using methods such as scHD4E, scDEA, Seurat, and others across all of the liver cancer datasets. However, we aimed further to validate the differentiability of these genes for liver cancer using a web-based database, the Gene Expression Profiling Interactive Analysis (GEPIA2) online platform (http://gepia2.cancer-pku.cn) (accessed on 20 November 2024). We assessed the differentiability of the identified genes in liver cancer by constructing boxplots for each gene using the GEPIA2 database. This database relies on data from the GTEx project and TCGA, which includes thousands of samples from various cancer types.

### 2.8. Liver Cancer Stage-Wise Relation to Hub Genes

To illustrate the effects of each hub gene on the development and prognosis across different stages of liver cancer, we constructed stage plots using the Gene Expression Profiling Interactive Analysis (GEPIA2) online platform (http://gepia2.cancer-pku.cn) (accessed on 20 November 2024) [29]. The stage plots revealed variations in gene expression across the different stages of liver cancer. If the *p*-values for these plots are ≤0.05, the data suggest that the genes are significantly associated with the progression of liver cancer at the respective stage.

### 2.9. Survival Analysis of hHub Genes for Liver Cancer

Survival analysis was conducted using three web-based databases, GEPIA2 [29], Kaplan-Meier (KM) Plotter [30], and UALCAN [31], to assess the risk associated with low and high expression of molecular signatures (genes) and evaluate the impact of hub genes on the prognosis and development of liver cancer. The use of these three databases allowed for more robust validation of the association between these genes and the survival of liver cancer patients. Overall and relapse-free survival analyses were performed using both GEPIA2 and KM Plotter. The survival plots displayed the hazard ratio (HR) with a 95% confidence interval and log-rank *p*-values for both GEPIA2 and KM Plotter. The UALCAN database focused on the effects of gene expression on patient survival, providing *p*-values. A *p*-value ≤ 0.05 was considered statistically significant.

### 2.10. Molecular Docking

Molecular docking analysis was conducted to propose validated, efficient candidate drugs to treat liver cancer by targeting the identified receptor proteins. The target proteins for the drugs were selected based on the hub-of-hub (hHub) genes, and approximately 300 drugs were considered as meta-drug agents. Additionally, 3D structures of both the meta-drug agents and receptor proteins were required for molecular docking analysis. We retrieved the 3D structures of the target proteins from the Protein Data Bank (PDB) [32] and the 3D structures of the meta-drug agents from the PubChem database [33].

Water molecules and ligand heteroatoms were removed to prepare the receptor proteins for molecular docking, and polar hydrogens were added [34]. A grid box was constructed to encompass the entire surface of the proteins. Using AutoDockTools, ligands were prepared by defining torsion trees and specifying the rotatable and non-rotatable bonds. The binding affinities between the drug agents and target proteins were computed using AutoDock Vina, with an exhaustiveness parameter set to 10.

## 3. Results

### 3.1. Identification of Common Differentially Expressed Genes

We applied all six methods to four real-world scRNA-seq liver cancer datasets and identified differentially expressed genes (DEGs) based on a significance cutoff of 0.05. Subsequently, we identified the common DEGs by taking the intersection of results from the two-hybrid methods, scDEA and scHD4E, and from all six methods (two hybrid and four individual). We also identified the common genes by considering all possible combinations of three datasets out of four for both the two-method and six-method analyses. The total number of combinations was eight.

The procedure is described in the methodology and illustrated in Figure 1. The numbers of common genes identified for each combination of datasets between scDEA and scHD4E were as follows: A ∩ B ∩ C = 165, A ∩ B ∩ D = 226, B ∩ D ∩ C = 1570, and A ∩ D ∩ C = 343. Similarly, for the intersection of all six methods, we identified 52, 114, 534, and 180 common genes for the same dataset combinations. A significant number of genes were eliminated from the analysis when only the intersection of six methods was used because some of the datasets had a small sample size. The results are presented in Figure 1.

### 3.2. Identification of Hub Genes Using STRING and Cytoscape

We constructed the protein−protein-interaction (PPI) network for each of the eight sets of common genes identified in the previous subsection using web-based tools. The PPI networks were organized and visualized using cytoHubba in Cytoscape, which allowed us to rank the important genes. We selected the top 15 hub genes for each of the eight gene sets. The results are presented in Table S4.

From the table, it is evident that several common genes are shared among the sets of hub genes. To ensure that none of the important genes were excluded from the study, we took the union of the sets of hub genes. This approach resulted in the identification of 62 genes, which were the focus of our subsequent analysis.

### 3.3. Protein−Protein-Interaction (PPI) Network and Selection of the Most Important Genes

The PPI network was constructed using the STRING database tools and Cytoscape software. 

**Figure 2:**
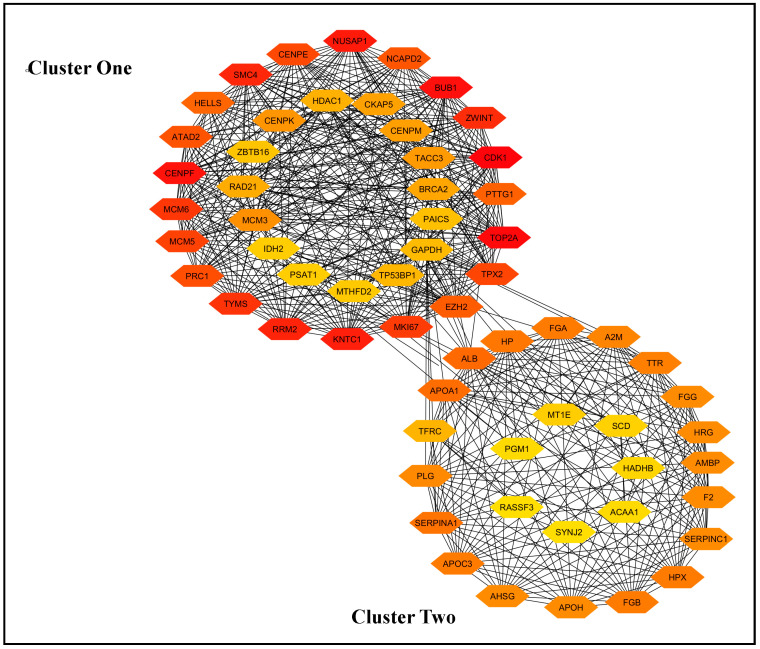
Protein–protein-interaction (PPI) network for 62 important genes, which are divided into two clusters. A deeper orange color indicates the genes have more interactions with the other genes.

The resulting network graph indicates that the genes are divided into two distinct clusters: cluster one and cluster two. Cluster one contains 35 genes, while cluster two consists of 27 genes (Figure 2). We selected the 25 hub genes for subsequent analysis using cytoHubba. Additionally, we identified 20 essential hub genes from cluster two. The 25 + 20 top-ranked genes are listed in Table S5.

### 3.4. Checking Differentiability of hHub Genes Using GEPIA2

We identified 25 hub genes from Cluster 1 and 20 hub genes from Cluster 2. The differential expression of these hub genes was analyzed in the context of liver cancer to determine their potential roles in prognosis, diagnosis, and disease progression.

**Figure 3:**
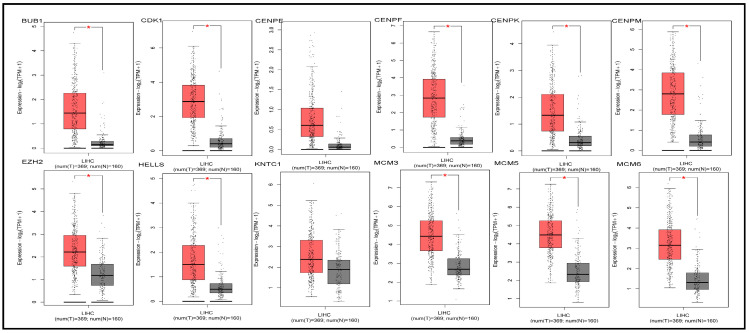
Boxplots of genes from the liver cancer case vs. control groups to show the differentiability of the following genes: BUB1, CDK1, CENPE, CENPF, CENPK, CENPM, EZH2, HELLS, KNTC1, MCM3, MCM5, MCM6. * indicates that a GENE is differentially expressed.

Genes exhibiting significant differential expression are hypothesized to influence the prognosis, diagnostic accuracy, and development of liver cancer. To assess differential expression, boxplots for all 45 hub genes were generated using the GEPIA online database (Figure 3 and Figures S1–S3). The results demonstrated that all 25 genes from Cluster 1 were significantly differentially expressed between liver cancer and control samples, thereby meeting the differential expression criteria established through our statistical analyses. A similar conclusion was reached for the 20 genes in Cluster 2.

### 3.5. Impact of Hub Genes on the Stage of Liver Cancer

**Figure 4:**
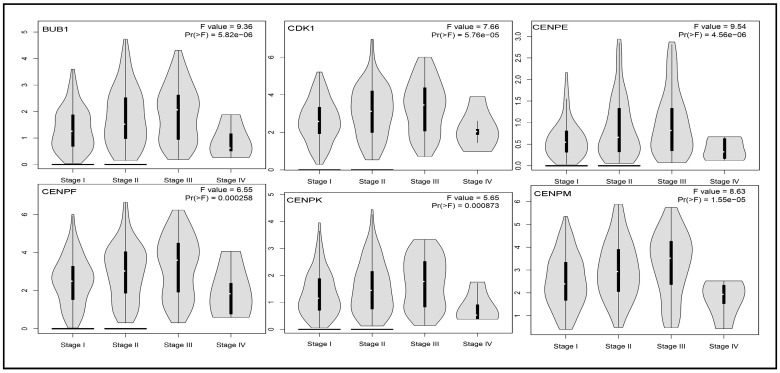
Violin plots of different stages of liver cancer representing the association of progression with the following genes: BUB1, CDK1, CENPE, CENPF, CENPK, CENPM.

Stage plots were generated for each hub gene in Clusters 1 and 2. All figures for Cluster 1 exhibited *p*-values ≤ 0.05, indicating that the expression of these hub genes significantly influences the progression of liver cancer across different stages (Figure 4 and Figures S4–S6). In Cluster 2, most genes were shown to have a significant impact, except for *A2M* (*p* = 0.305), which showed no notable association with liver cancer stage progression (Figures S7–S9).

### 3.6. GO and Pathway Analysis of Hub Genes

For gene ontology (GO) and pathway analysis, we utilized four web-based tools: DAVID, STRING, Enrichr, and WebGestalt. The top-ranked GO terms were selected when at least two tools identified them as significant based on *p*-values. Table 2 presents the gene ontology terms for Biological Processes (BPs) and KEGG pathways, along with the corresponding hub genes implicated in these processes. These biological processes and pathways are associated with liver cancer prognosis, diagnosis, and development, as supported by references listed in Table 2.

The top-ranked Molecular Functions (MFs) and Cellular Components (CCs) are provided in Table S1 and summarized below:

**Molecular Functions:** protein binding [35,36], identical protein binding [37], endopeptidase inhibitor activity [38], serine-type endopeptidase inhibitor activity [39], single-stranded DNA binding [40], ATP binding [41,42], and protein homodimerization activity [43] (detailed in Table S1).

**Cellular Components:** enzyme inhibitor activity [44,45], blood microparticles [46,47], platelet alpha granule lumen [48], extracellular exosome [49], condensed chromosome centromeric region [50], endoplasmic reticulum lumen [51], chromosome [52], collagen-containing extracellular matrix [53], and spindle [54] (detailed in Table S1).

We also incorporated Reactome and WikiPathways data, which validated the associations between hub genes and liver cancer. These pathways included the following:

**Reactome Pathways:** cell cycle, mitosis [55]; platelet degranulation [56]; platelet activation, signaling, and aggregation [57]; regulation of insulin-like growth factor transport and uptake by insulin-like growth factor binding proteins [58]; mitotic prometaphase [59]; and cell cycle checkpoints [60] (detailed Table S2).

**WikiPathways:** folate metabolism [61], complement system [62], gastric cancer network 1 [63], selenium micronutrient network [64], and retinoblastoma gene in cancer [65] (detailed in Table S2).

**Table 2:** Top common gene ontology (BPs) and KEGG pathways derived from the four databases.

Category	GO ID	Terms and References	Associated Gene Count				Common Associated Genes from at Least Two Databases
			DAVID	STRING	Enrichr	WebGestalt	
Biological Process	GO:0051301	cell division [66]	14	16	-	-	BUB1, PTTG1, TPX2, ZWINT, CENPE, CENPF, CDK1, HELLS, KNTC1, MCM5, NCAPD2, PRC1, SMC4, TACC3
	GO:0042730	fibrinolysis [67]	06	06	05	07	F2, FGA, FGB, FGG, HRG, PLG
	GO:0000070	mitotic sister chromatid segregation [68]	05	08	09	-	ZWINT, CENPK, KNTC1, NUSAP1, SMC4
	GO:0031639	plasminogen activation [69]	04	04	03	-	FGA, FGB, FGG, APOH
	GO:0030193	regulation of blood coagulation [70]	04	08	02	-	APOH, F2, HRG, SERPINC1
	GO:0007596	blood coagulation [71]	06	09	-	-	F2, FGA, FGB, PLG, SERPINA1, SERPINC1
	GO:0006953	acute-phase response [72]	06	06	-	-	AHSG, A2M, F2, HP, SERPINA1, TFRC
	GO:0051918	negative regulation of fibrinolysis	04	04	02	-	PLG, F2, HRG, APOH
	GO:0030168	platelet activation [73]	05	05	-	-	F2, FGA, FGB, HRG, FGG
	GO:0051382	kinetochore assembly [74]	03	04	03	-	CENPE, CENPF, CENPK, KNTC1
	GO:0051383	kinetochore organization	-	05	02	-	CENPE, CENPF
	GO:0007094	mitotic spindle assembly checkpoint signaling [75]	04	04	04	-	BUB1, ZWINT, CENPF, KNTC1
	GO:0072378	blood coagulation, fibrin clot formation [76]	03	05	03	-	FGA, FGG, FGB
	GO:1900026	positive regulation of substrate adhesion-dependent cell spreading	04	04	04	-	FGA, FGG, FGB, APOA1
	GO:0090277	positive regulation of peptide hormone secretion [77]	03	-	03	-	FGA, FGG, FGB, APOA1
	GO:0034508	centromere complex assembly [78]	-	05	02	-	CENPE, CENPF
	GO:0030195	negative regulation of blood coagulation	-	07	06	-	FGA, FGB, FGG, PLG, F2, APOH
	GO:0140014	mitotic nuclear division [79]	-	09	05	13	NCAPD2, CENPE, CENPK, PRC1, NUSAP1, TPX2, ZWINT, KNTC1, SMC4
	GO:0000280	nuclear division	-	12	-	14	ZWINT, BUB1, PRC1, PTTG1, TOP2A, SMC4, TPX2, CENPE, KNTC1, NUSAP1, NCAPD2
	GO:1903047	mitotic cell cycle process [55]	-	16	-	21	NCAPD2, KNTC1, MCM3, MCM6, PRC1, CENPF, SMC4, TACC3, TPX2, BUB1, CDK1, ZWINT, CENPE, NUSAP1, EZH2
			Associated Gene Count				Associated Common Genes
Database	ID	Pathways	DAVID	STRING	Enrichr	WebGestalt	
KEGG	hsa04610	complement and coagulation cascades [80]	08	08	08	08	A2M, F2, FGA, FGB, FGG, PLG, SERPINA1, SERPINC1
	hsa04110	cell cycle [81]	06	06	06	05	BUB1, PTTG1, CDK1, MCM3, MCM5, MCM6
	hsa03030	DNA replication [82]	03	03	03	03	MCM3, MCM5, MCM6
	hsa04611	platelet activation [83]	04	04	04	03	F2, FGA, FGB, FGG
	hsa04979	cholesterol metabolism [84]	03	03	03	03	APOA1, APOC3, APOH
	hsa04114	oocyte meiosis	03	-	03	03	BUB1, PTTG1, CDK1
		coronavirus disease [85]	04	-	04		F2, FGA, FGB, FGG
	hsa04115	p53 signaling pathway [86]	-	-	02	02	CDK1, RRM2

### 3.7. Prognostic Power of Hub Genes

We performed survival analysis on 45 hub genes (25 from Cluster 1 and 20 from Cluster 2) using three databases: GEPIA, UALCAN, and KM Plotter.

This multi-database approach was employed to strengthen the evidence for the prognostic significance of these genes. Both GEPIA and KM Plotter provided analyses for overall survival and relapse-free survival, offering comprehensive insights into the prognostic power of the identified hub genes. The results for overall survival and relapse-free survival, as illustrated in the plots generated by GEPIA and KM Plotter for Cluster 1 hub genes, are presented in Table S6, along with Figure 5, Figure 6 and Figures S10–S23. Additionally, the overall survival plots generated by UALCAN are provided in Figures S24–S27. Relevant plot metrics, including hazard ratio (HR), *p*(HR), log-rank *p*-values, and overall *p*-values, are detailed in Table S6 for the Cluster 1 hub genes.

Analysis of the figures and tables reveals that the expression levels of all hub genes significantly influence liver cancer prognosis, diagnosis, and development. Specifically, lower expression levels of all Cluster 1 hub genes were associated with an increased survival probability in patients, highlighting their critical role in liver cancer progression. Similarly, for the Cluster 2 hub genes, overall and relapse-free survival plots were generated, and the findings are summarized in Table S3. The results indicate that several hub genes (*A2M*, *AHSG*, *ALB*, *F2*, *HP*, and *SERPINA1*) are not significant in survival analysis. However, the remaining Cluster 2 genes showed significant associations: higher expression levels of *AMBP*, *APOA1*, *APOC3*, *APOH*, *FGA*, *FGB*, *FGG*, *HPX*, *HRG*, *PLG*, *SERPINC1*, and *TTR* were linked to improved patient survival. Conversely, lower expression levels of *GAPDH* and *TFRC* significantly increased survival probability, underscoring their prognostic importance.

### 3.8. Molecular Docking for Drug Repurposing Guided by Biomarker Genes

We identified 24 hHubGs (TOP2A, CDK1, BUB1, CENPF, NUSAP1, KNTC1, RRM2, SMC4, ZWINT, TYMS, MCM6, MKI67, MCM5, CENPE, TPX2, PRC1, NCAPD2, EZH2, PTTG1, HELLS, MCM3, CENPK, TACC3, and CENPM) from Cluster 1 and 12 hHubGs (AMBP, APOC3, FGA, FGB, GAPDH, HPX, FGG, HRG, PLG, SERPINC1, and TFRC) from Cluster 2 as potential hHub genes based on their statistical significance in survival analysis. One gene from Cluster 1 and eight genes from Cluster 2 were excluded from further analysis.

We obtained the 3D structures of these 24 + 12 target protein receptors from the Protein Data Bank (PDB) [34]. Additionally, 300 candidate cancer drugs were compiled from the Cancer Research UK website and a literature review [87,88,89], and their 3D structures were downloaded from PubChem [33]. Molecular docking was performed between the 36 target proteins and the 300 drug candidates to calculate binding scores for each target protein/drug pair. A subset of candidate drugs was identified by ranking the target proteins based on the row averages of binding scores and sorting drugs using column averages. We selected the 10 top-ranked drugs for the 24 hHubGs from Cluster 1 and 12 hHubGs from Cluster 2.

**Figure 7:**
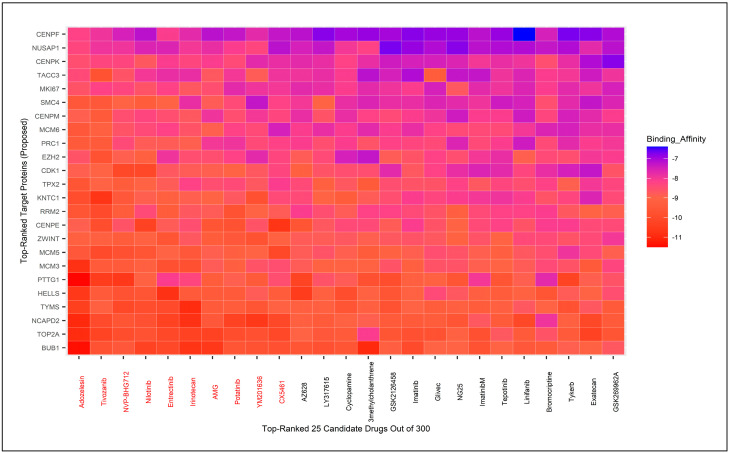
The 25 top-ranked meta-drugs against ordered target proteins based on binding affinity score for cluster-one hHubGs.

Interestingly, the same 10 drugs (*Adozelesin*, *Tivozanib*, *NVP-BHG712*, *Nilotinib*, *Entrectinib*, *Irinotecan*, *AMG*, *Ponatinib*, *YM201636*, *and CX5461*) emerged as top-ranked candidates in both scenarios (Figure 7 and Figures S28–S30). These 10 drugs have been considered as potential candidates; however, further validation is required to confirm their therapeutic efficacy. We searched the literature to identify the hub genes named in multiple previously published articles [1,2,9,10,11,12,90,91,92,93,94]. A total of 42 hub genes were listed from about 15 published papers. Using STRING, Cytoscape, and cytoHubba, we ranked the 42 genes, and the top 20 were selected for molecular docking with drug agents.

**Figure 8:**
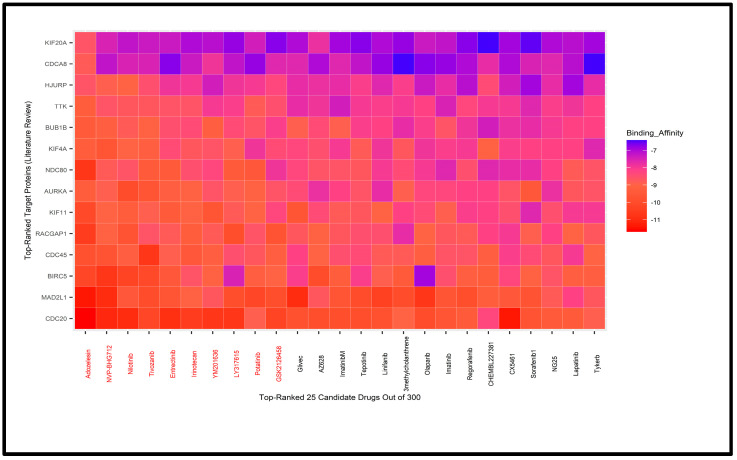
The 25 top-ranked meta-drugs against ordered target proteins based on binding affinity score for published HubGs.

However, the 3D structures of six ranked genes were not available in PDB. Consequently, we used 14 top ranked genes as receptor proteins and performed molecular docking analysis with 300 meta-drug agents (Figure 8 and Figure S31). We noticed that there were eight drugs in common between the selected drugs for our hHubGs (24 + 12 genes) and 14 HubGs from the published HubGs. The published top ranked hub genes were as follows: *AURKA*, *BIRC5*, *BUB1B*, *CDC20*, *CDC25*, *CDCA8*, *HJURP*, *KIF4A*, *KIF11*, *KIF20A*, *MAD2L1*, *NDC80*, *RACGAP1* and *TTK*. Finally, after validation, we suggested eight drugs (Adozelesin, Tivozanib, NVP-BHG712, Nilotinib, Entrectinib, Irinotecan, Ponatinib, YM201636) as our candidate drugs.

## 4. Discussion

Although various studies have been conducted to identify disease-related genes and pathways in liver cancer, early detection and timely initiation of treatment remain significant challenges due to the complex mechanisms of cancer initiation and progression [2]. Consequently, the mortality rate of liver cancer has gradually increased over recent years, particularly in developing countries. Therefore, it is imperative to identify sensitive and specific genes that accurately track liver cancer progression, enabling a detailed understanding of the molecular mechanisms underlying the prognosis and treatment of liver cancer [92]. According to our literature review, previous studies on the identification of key genes in liver cancer have predominantly utilized bulk RNA-seq or microarray data (2,9,10,68,90), but the results have been inconsistent in terms of accurate detection of DEGs. A study based on microarray data identified five genes (CDK1, CDC20, CCNB1, CENPF, and MAD2L1) related to the tumor stage, early diagnosis, and poor outcomes [91]. CDKN2A, RB1, TP53, and PTEN were identified as key genes in another study [9]. BUB1B, TOP2A, KIF23, UBE2C, KIF15, CDC20, PLK1, HJURP, BUB1, and DLGAP5 were selected by another bioinformatics analysis based on microarray data [1]. TTK, NCAPG, TOP2A, CCNB1, CDK1, PRC1, RRM2, UBE2C, ZWINT, CDKN3, AURKA, and RACGAP1 were identified as hub genes in a study conducted by Wu M et al. [2]. Therefore, it is clear that results based on RNA-seq or microarray data were not consistent, which indicates the necessity of differential expression analysis of scRNA-seq data using modern methods.

In this study, we conducted an integrated bioinformatics analysis using four scRNA-seq datasets (GSE98638, GSE146409, GSE202069, GSE189935), applying two hybrid and four top-performing individual methods. The analysis procedures are illustrated in Figure 1 and were designed to capture all potential genes. Initially, we identified differentially expressed genes with adjusted *p*-values ≤ 0.05 and found intersecting genes for the two hybrid methods and six methods (including hybrid and individual methods) across all four scRNA-seq datasets. We selected intersecting genes from three out of four datasets, resulting in eight sets of intersecting genes. Using STRING, Cytoscape, and cytoHubba, we identified 15 hub genes from each set of intersecting genes, yielding 62 hub genes by taking the union of the eight sets. These 62 genes were naturally divided into two clusters by Cytoscape networking (Figure 2). From cluster one, we selected 25 hub-of-hub genes (hHubGs), and from cluster two, 20 hHubGs. The two sets of hHubGs were as follows: {*TOP2A*, *CDK1*, *BUB1*, *CENPF*, *NUSAP1*, *KNTC1*, *RRM2*, *SMC4*, *ZWINT*, *TYMS*, *MCM6*, *MKI67*, *MCM5*, *CENPE*, *TPX2*, *PRC1*, *ATAD2*, *NCAPD2*, *EZH2*, *PTTG1*, *HELLS*, *MCM3*, *CENPK*, *TACC3*, *CENPM*} and {*ALB*, *APOA1*, *SERPINA1*, *HP*, *HPX*, *FGB*, *APOC3*, *FGA*, *TTR*, *A2M*, *PLG*, *AHSG*, *F2*, *FGG*, *HRG*, *SERPINC1*, *APOH*, *AMBP*, *TFRC*, *GAPDH*}. Many of the hHubGs included in these sets were not identified in previous liver cancer studies but play vital roles in liver cancer development and could be used to target treatment (as discussed in the results section). We identified approximately 42 hHubGs from 10 published articles based on liver cancer data, with the main goal of these articles being the identification of hub genes and different pathways. We selected the top 20 hub genes from published hub genes (hHubGs) using the PPI network, as follows: {*CCNB2*, *CDC20*, *KIF11*, *ASPM*, *BIRC5*, *NDC80*, *CDCA5*, *CDCA8*, *SPC25*, *KIF4A*, *KIF20A*, *CDC45*, *RACGAP1*, *HJURP*, *BUB1B*, *MAD2L1*, *AURKB*, *TTK*, *KIF2C*, *AURKA*}. We validated our study’s hHubGs for differential expression using boxplots generated with GEPIA2 (Figure 3 and Figures S1–S3) and constructed stage plots to assess the impact of these genes on the development of liver cancer (Figure 4 and Figures S4–S9) (briefly described in the subsections Checking Differentiability of Hub Genes Using GEPIA and Impact of Hub Genes on the Stage of Liver Cancer).

The differentially expressed hHubGs are robustly associated with various biological processes, molecular functions, and cellular components that accelerate liver cancer progression (Table 2 and Table S1). KEGG, Wiki, and Reactome pathway analyses identified several pathways linked to liver cancer (Table 2 and Table S2). This information was validated with references in the subsection GO and Pathway Analysis of Hub Genes. We also assessed the prognostic power of hHubGs through survival analysis using the GEPIA2, UALCAN, and KM plotter web tools. The survival analysis indicated that the ATAD2 gene in cluster one was not statistically significant, while only 12 hHubGs from cluster two were statistically significant. These are as follows: *AMBP*, *APOC3*, *FGA*, *FGB*, *GAPDH*, *HPX*, *FGG*, *HRG*, *PLG*, *SERPINC1*, and *TFRC*. We used only the hHubGs from clusters one and two with prognostic power for liver cancer to conduct molecular docking. A total of 24 + 12 hHubGs were used for molecular docking. We validated 24 genes from cluster one and 12 genes for cluster two from the literature review. OIP5, ASPM, NUSAP1, UBE2C, CCNA2, and KIF20A were the hub genes identified by Li et al. [95]. KNTC1, SMC4, TYMS, MCM6, MKI67, MCM5, CENPE, TPX2, NCAPD2, EZH2, PTTG1, HELLS, MCM3, TACC3, and CENPM were identified as hub genes from cluster one through different studies [96,97,98,99,100,101,102,103,104,105,106,107,108,109,110]. AMBP, APOC3, FGA, FGB, HPX, FGG, HRG, PLG, SERPINC1, and TFRC were associated with liver cancer [111,112,113,114,115,116,117,118,119,120]. KNTC1 knockdown inhibits the proliferation and metastases of liver cancer.

We selected 10 candidate drugs out of 300 based on binding affinity scores between protein receptors and meta-drug agents. These 10 candidate drugs were Adozelesin, Tivozanib, NVP-BHG712, Nilotinib, Entrectinib, Irinotecan, AMG, Potatinib, YM201636, and CX5461 (Figure 7 and Figures S28–S30). Our study also conducted molecular analyses to relate the published hHubGs to the candidate drugs (Figure 8 and Figure S31). Out of the 10 drugs, eight were both among the top-ranked drugs identified by our analysis and identified in various published analyses. We also validated the drugs by conducting a literature review. Adozelesin is used to treat colon cancer [121]. Tivozanib, NVP-BHG712, Nilotinib, ponatinib and YM201636 are used for hepatocellular carcinoma [122,123,124,125]. Irrinotecan is used as a key chemotherapeutic drug for colorectal cancer. Entrectinib is a new multi-target inhibitor for cancer therapy [126]. Therefore, after validation, Adozelesin, Tivozanib, NVP-BHG712, Nilotinib, Entrectinib, Irinotecan, Potatinib, and YM201636 are suggested as candidate drugs for liver cancer based on our study.

## 5. Conclusions

Liver cancer is one of the most prevalent cancers and the second leading cause of cancer-related deaths, imposing a significant financial burden worldwide, particularly in developing countries.

This study aimed to identify potential hub genes and perform functional analysis using these genes, as well as to suggest drugs through molecular docking based on analysis of scRNA-seq liver cancer data. We employed two recently developed hybrid methods and four top-performing individual methods on four scRNA-seq datasets to identify differentially expressed genes. Our study identified 25 hub-of-hub genes (hHubGs) and 20 hHubGs from two gene clusters that naturally formed using the PPI network, ultimately validating 36 (24 + 12) hHubGs. Functional and pathway analyses indicated that the functions/pathways identified by hHubGs are closely associated with liver cancer. The study suggested 10 candidate drugs out of 300 cancer meta-drugs through molecular docking based on the binding affinity scores of 36 receptor proteins and validated these 10 drugs using the top-ranked 14 published receptor proteins. Consequently, Adozelesin, Tivozanib, NVP-BHG712, Nilotinib, Entrectinib, Irinotecan, Potatinib, and YM201636 are recommended as candidate drugs for liver cancer based on our study.

## Figures and Tables

**Figure 5:**
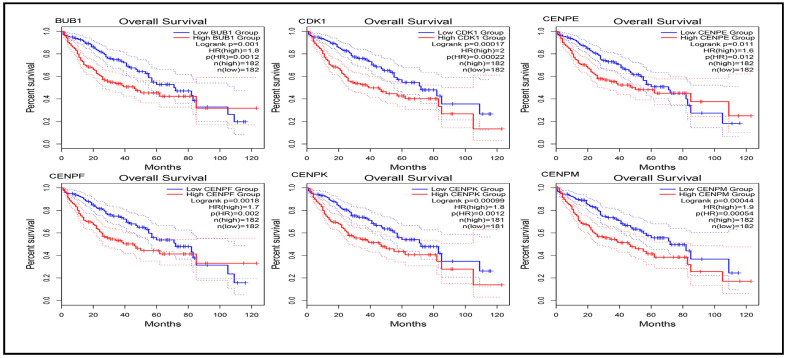
Overall survival plot to investigate the effect of low and high expression of the following hub genes: BUB1, CDK1, CENPE, CENPF, CENPK, CENPM; generated by GEPIA.

**Figure 6:**
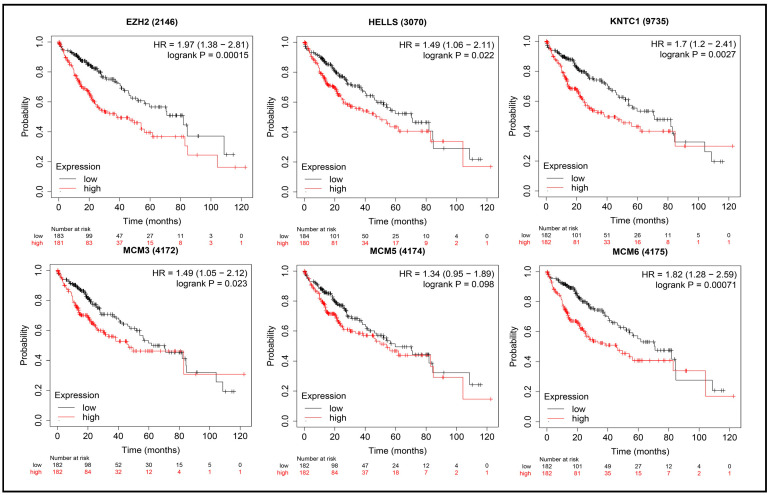
Overall survival plot to investigate the effect of low and high expression of the following hub genes: EZH2, HELLS, KNTC1, MCM3, MCM5, MCM6; generated by KM plotter.

**Table 1:** Description of curated liver cancer datasets.

Accession No.	Compared Cell Subset	Number of Samples per Group	Number of Features	Organism	Data Source
GSE98638	CD8+ T cells from adjacent normal liver tissues (NTC) vs. CD8+ T cells from liver tumor (TTC)	412 vs. 777	9288	Human	GEO
GSE146409	Non-malignant liver tumor vs. malignant liver tumor	12 vs. 63	15061	Human	GEO
GSE202069	Non-tumor vs. tumor patient	25 vs. 41	24492	Human	GEO
GSE189935	Adjacent normal tissues vs. tumor tissues	987 vs. 2199	15782	Human	GEO

## Data Availability

Data were downloaded from NCBI Gene Expression Omnibus (GEO) databases (https://www.ncbi.nlm.nih.gov/geo) (accessed on 15 October 2024) and these have been briefly described in the data source section.

## Supplementary Materials

The following supporting information can be downloaded at: https://www.mdpi.com/article/10.3390/biology14040431/s1, Table S1: Top common gene ontology (Molecular function and Cellular component) from four databases. Table S2: Top Common reactome and wiki pathways from four databases. Table S3: Survival plot related values generated form GEPA, KM plotter and UALCAN databases for cluster two hub genes. Table S4: Hub genes identification for different intersection of datasets for two and six methods using String and Cytoscape. Table S5: hHub Genes for cluster one and cluster two. Table S6: Survival plot related values generated form GEPA, KM plotter and UALCAN databases for cluster one hub genes. Figure S1: Boxplot between case vs control group for liver cancer for the genes: A2M, AHSG, ALB, AMBP, APOA1, APOC3, APOH, F2, FGA, FGB. Figure S2: Boxplot between case vs control group for liver cancer for the genes: FGG, GAPDH, HP, HPX, HRG, PLG, SERPINA1, SERPINC1, TFRC, TTR. Figure S3: Boxplot between case vs control group for liver cancer for the genes: ATAD2, MKI67, NCAPD2, NUSAP1, PRC1, PTTG1, RRM2, SMC4, TACC3, TOP2A, TPX2, TYMS, ZWINT. Figure S4: Violin plots of different stages of liver cancer for the Genes: EZH2, HELLS, KNTC1, MCM3, MCM5, MKI67. Figure S5: Violin plots of different stages of liver cancer for the Genes: NCAPD2, NUSAP1, PRC1, PTTG1, RRM2, SMC4. Figure S6: Violin plots of different stages of liver cancer for the Genes: TACC3, TOP2A, TPX2, TYMS, MCM6, ZWINT, ATAD2. Figure S7: Violin plots of different stages of liver cancer for the Genes: A2M, AHSG, ALB, AMBP, APOA1, APOC3. Figure S8: Violin plots of different stages of liver cancer for the Genes: APOH, F2, FGA, FGB, FGG, GAPDH. Figure S9: Violin plots of different stages of liver cancer for the Genes: HP, HPX, HRG, PLG, SERPINA1, SERPINC1, TFRC, TTR. Figure S10: Overall survival plot to investigate the effect of low and high expression for the following hub genes: EZH2, HELLS, KNTC1, MCM3, MCM5, MCM6 generated by GEPIA. Figure S11: Overall survival plot to investigate the effect of low and high expression for the following hub genes: MKI67, NCAPD2, NUSAP1, PRC1, PTTG1, RRM2 generated by GEPIA. Figure S12: Overall survival plot to investigate the effect of low and high expression for the following hub genes: SMC4, TACC3, TOP2A, TPX2, TYMS, ZWINT and ATAD2 generated by GEPIA. Figure S13: Relapse free survival plot to investigate the effect of low and high expression for the following hub genes: BUB1, CDK1, CENPE, CENPF, CENPK and CENPM generated by GEPIA. Figure S14: Relapse free survival plot to investigate the effect of low and high expression for the following hub genes: EZH2, HELLS, KNTC1, MCM3, MCM5, MCM6 generated by GEPIA. Figure S15: Relapse free survival plot to investigate the effect of low and high expression for the following hub genes: MKI67, NCAPD2, NUSAP1, PRC1, PTTG1, RRM2 generated by GEPIA. Figure S16: Relapse free survival plot to investigate the effect of low and high expression for the following hub genes: SMC4, TACC3, TOP2A, TPX2, TYMS, ZWINT and ATAD2 generated by GEPIA. Figure S17: Overall survival plot to investigate the effect of low and high expression for the following hub genes: BUB1, CDK1, CENPE, CENPF, CENPK, CENPM generated by KM plotter. Figure S18: Overall survival plot to investigate the effect of low and high expression for the following hub genes: MKI67, NCAPD2, NUSAP1, PRC1, PTTG1, RRM2 generated by KM plotter. Figure S19: Overall survival plot to investigate the effect of low and high expression for the following hub genes: SMC4, TACC3, TOP2A, TPX2, TYMS, ZWINT, ATAD2 generated by KM plotter. Figure S20: Relapse free survival plot to investigate the effect of low and high expression for the following hub genes: BUB1, CDK1, CENPE, CENPF, CENPK, CENPM generated by KM plotter. Figure S21: Relapse free survival plot to investigate the effect of low and high expression for the following hub genes: EZH2, HELLS, KNTC1, MCM3, MCM5, MCM6 generated by KM plotter. Figure S22: Relapse free survival plot to investigate the effect of low and high expression for the following hub genes: MKI67, NCAPD2, NUSAP1, PRC1, PTTG1, RRM2 generated by KM plotter. Figure S23: Relapse free survival plot to investigate the effect of low and high expression for the following hub genes: SMC4, TACC3, TOP2A, TPX2, TYMS, ZWINT, ATAD2 generated by KM plotter. Figure S24: Relapse free survival plot to investigate the effect of low and high expression for the following hub genes: BUB1, CDK1, CENPE, CENPF, CENPK, CENPM. Figure S25: Relapse free survival plot to investigate the effect of low and high expression for the following hub genes: EZH2, HELLS, KNTC1, MCM3, MCM5, MCM6. Figure S26: Relapse free survival plot to investigate the effect of low and high expression for the following hub genes: MKI67, NCAPD2, NUSAP1, PRC1, PTTG1, RRM2. Figure S27: Relapse free survival plot to investigate the effect of low and high expression for the following hub genes: SMC4, TACC3, TOP2A, TPX2, TYMS, ZWINT, ATAD2. Figure S28: Top ranked 50 meta-drugs against ordered target protein based on binding affinity score for cluster one hHubGs. Figure S29: Top ranked 50 meta-drugs against ordered target protein based on binding affinity score for cluster two hHubGs. Figure S30: Top ranked 25 meta-drugs against ordered target protein based on binding affinity score for cluster two hHubGs. Figure S31: Top ranked 25 meta-drugs against ordered target protein based on binding affinity score for cluster published hHubGs.
