# Supplementary material for: Discovery of Genomic Targets and Therapeutic Candidates for Liver Cancer Using Single-Cell RNA Sequencing and Molecular Docking
Source: Biology (Basel). 2025 Apr 17;14(4):431. doi: 10.3390/biology14040431 (PMC12024973; doi:10.3390/biology14040431)
Supplement: Supplementary file 1 [file biology-14-00431-s001.zip › Supplementary Figure.pptx]

## Slide 1
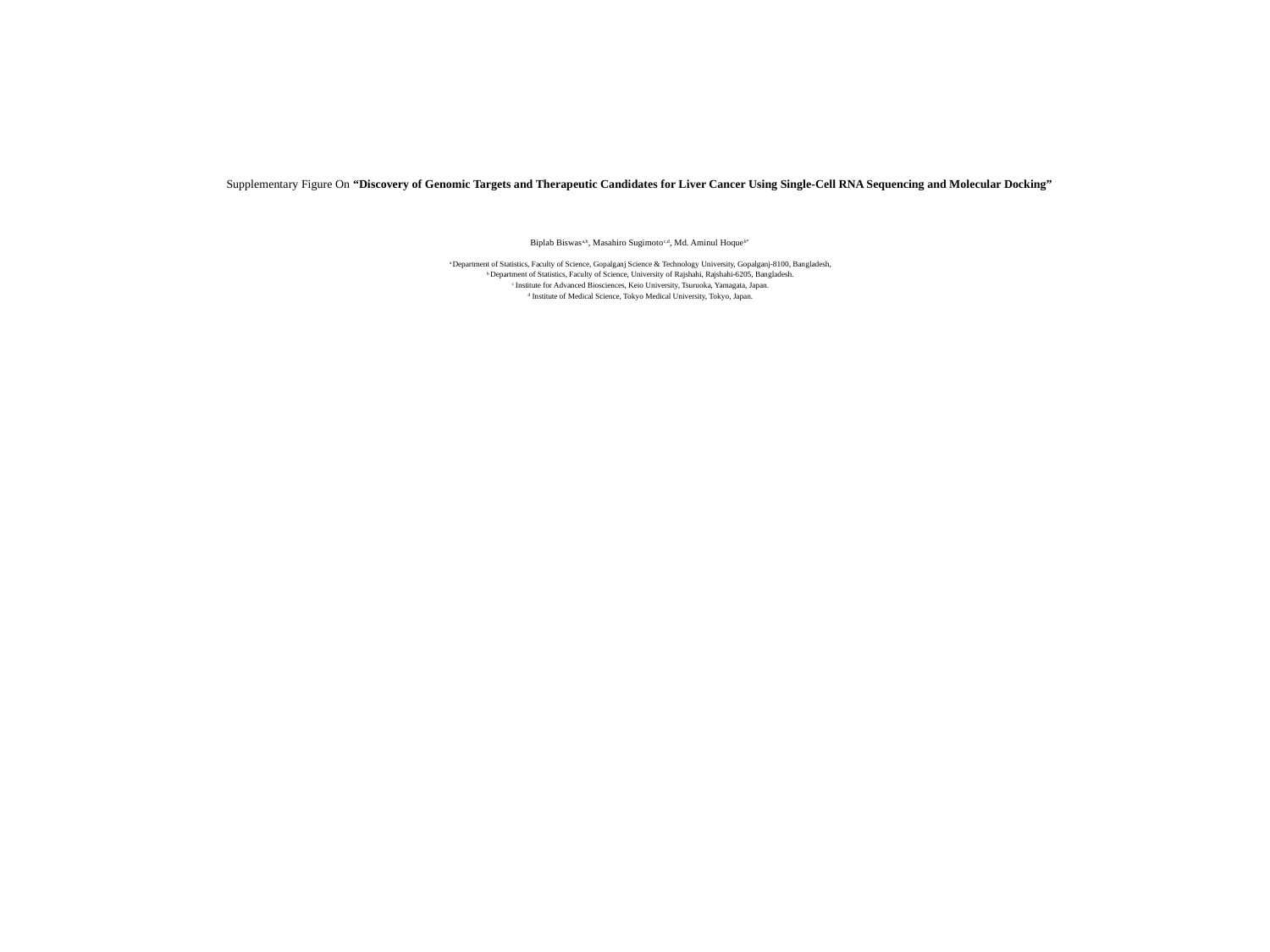

# Supplementary Figure On “Discovery of Genomic Targets and Therapeutic Candidates for Liver Cancer Using Single-Cell RNA Sequencing and Molecular Docking” Biplab Biswasa,b, Masahiro Sugimotoc,d, Md. Aminul Hoqueb*   a Department of Statistics, Faculty of Science, Gopalganj Science & Technology University, Gopalganj-8100, Bangladesh, b Department of Statistics, Faculty of Science, University of Rajshahi, Rajshahi-6205, Bangladesh. c Institute for Advanced Biosciences, Keio University, Tsuruoka, Yamagata, Japan. d Institute of Medical Science, Tokyo Medical University, Tokyo, Japan.

## Slide 2
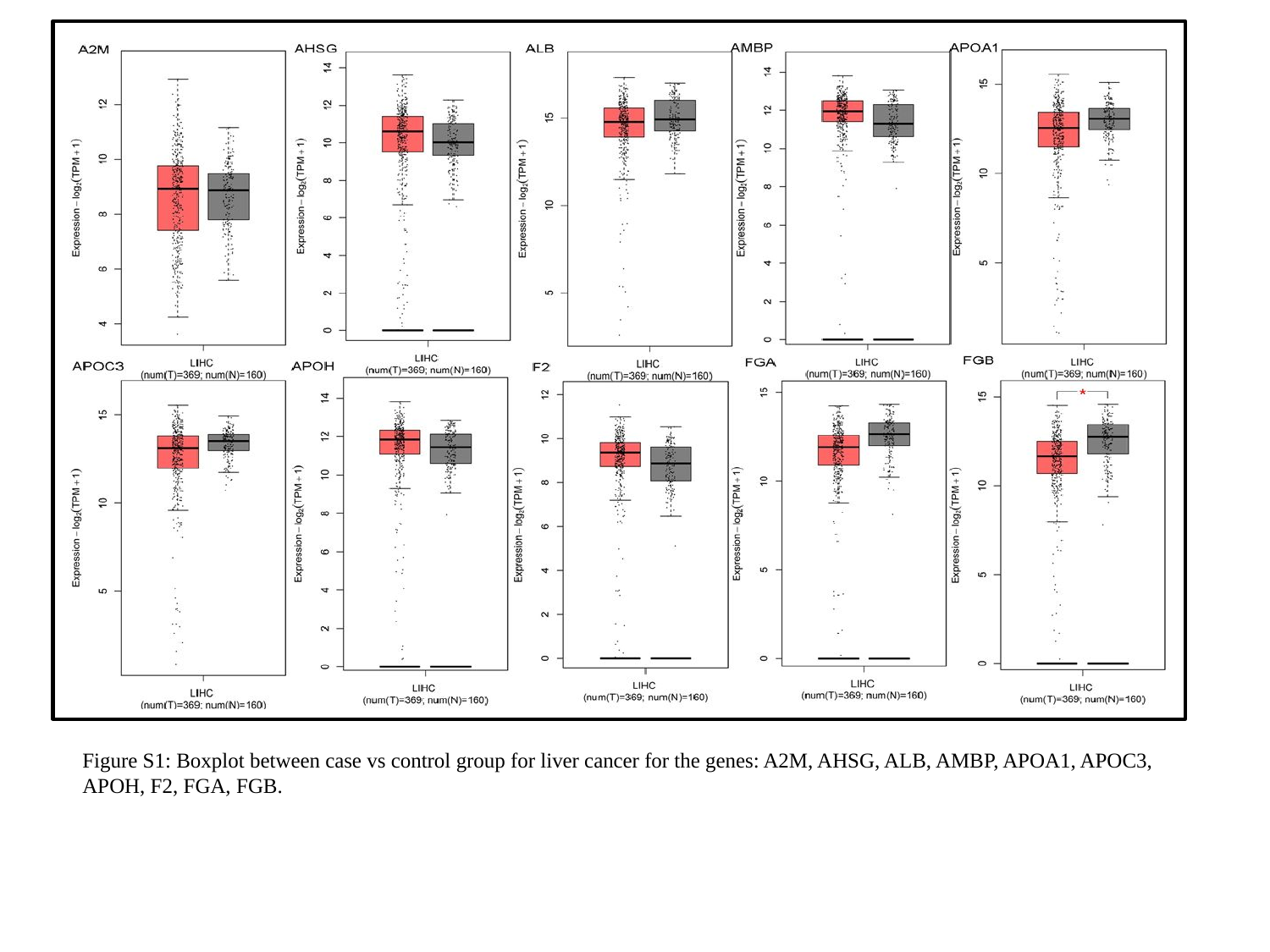

Figure S1: Boxplot between case vs control group for liver cancer for the genes: A2M, AHSG, ALB, AMBP, APOA1, APOC3, APOH, F2, FGA, FGB.

## Slide 3
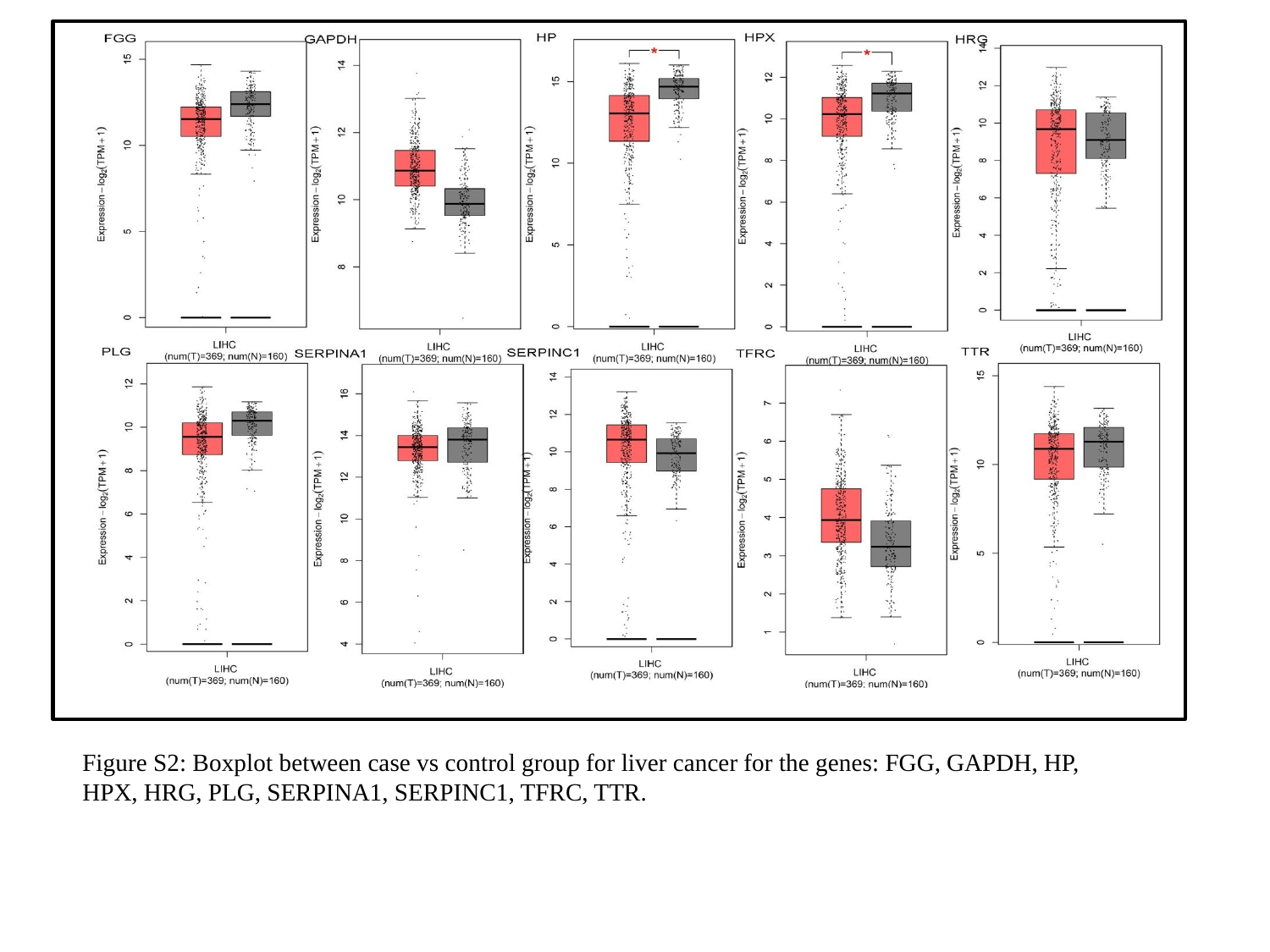

Figure S2: Boxplot between case vs control group for liver cancer for the genes: FGG, GAPDH, HP, HPX, HRG, PLG, SERPINA1, SERPINC1, TFRC, TTR.

## Slide 4
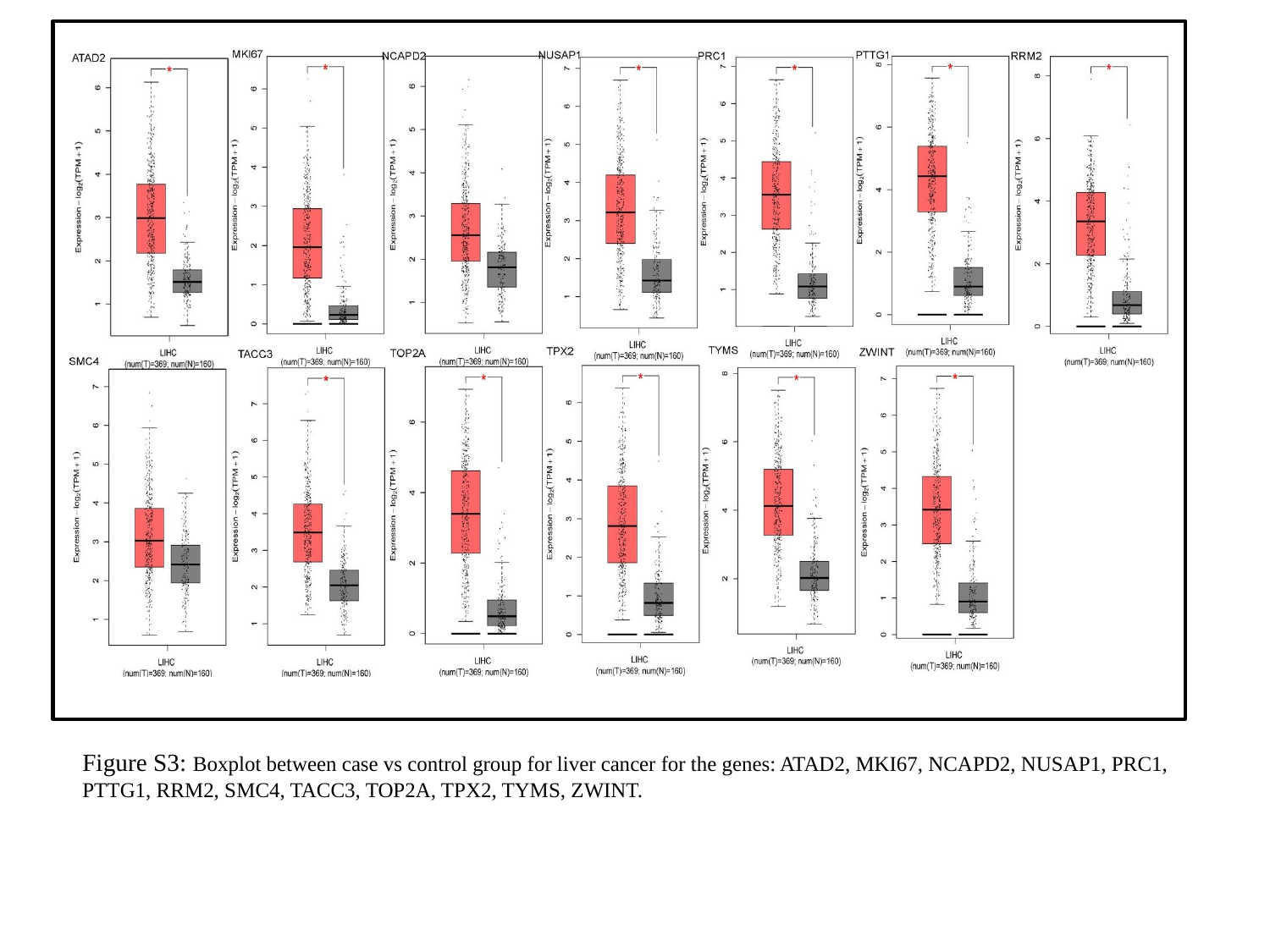

Figure S3: Boxplot between case vs control group for liver cancer for the genes: ATAD2, MKI67, NCAPD2, NUSAP1, PRC1, PTTG1, RRM2, SMC4, TACC3, TOP2A, TPX2, TYMS, ZWINT.

## Slide 5
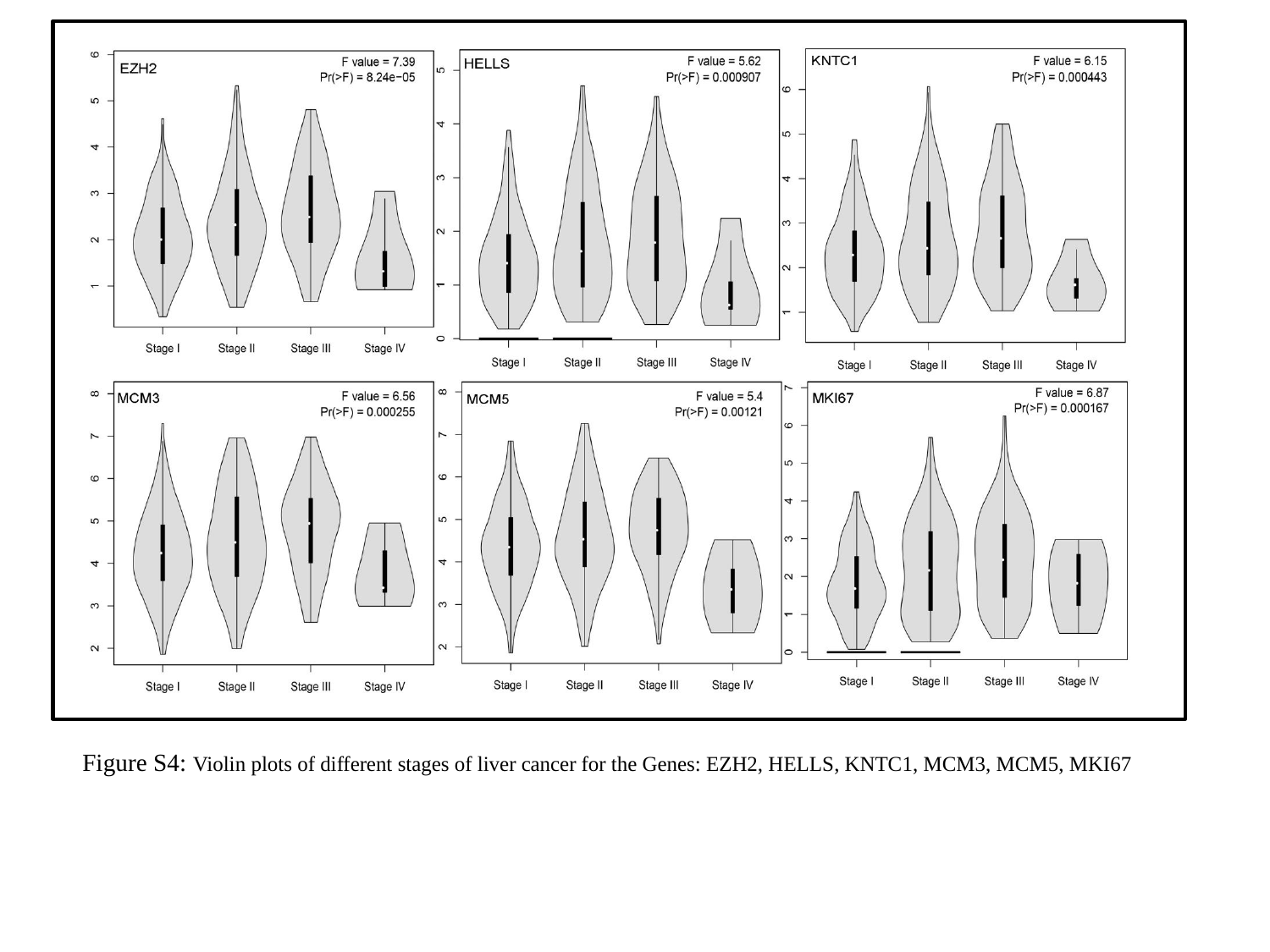

Figure S4: Violin plots of different stages of liver cancer for the Genes: EZH2, HELLS, KNTC1, MCM3, MCM5, MKI67

## Slide 6
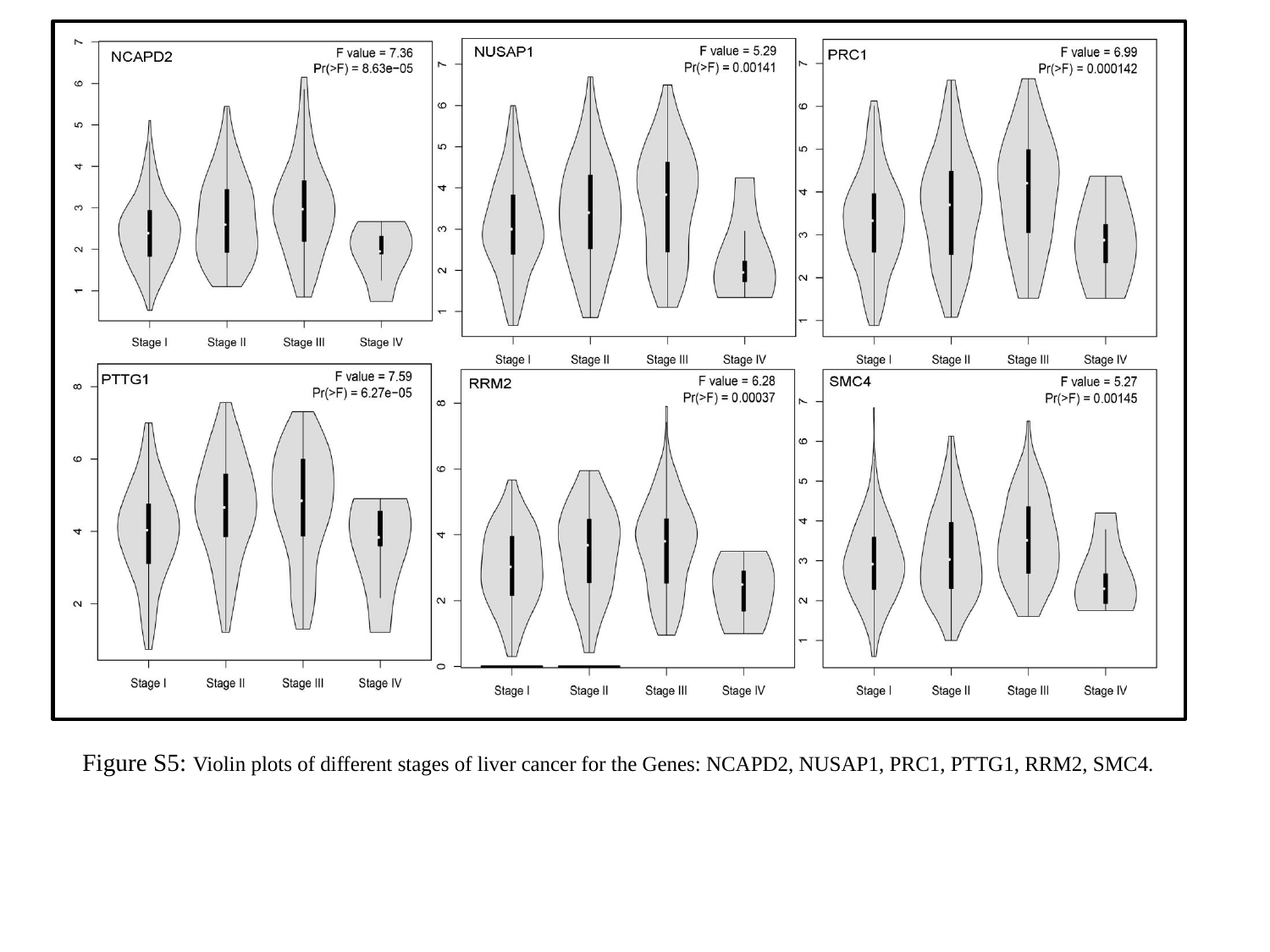

Figure S5: Violin plots of different stages of liver cancer for the Genes: NCAPD2, NUSAP1, PRC1, PTTG1, RRM2, SMC4.

## Slide 7
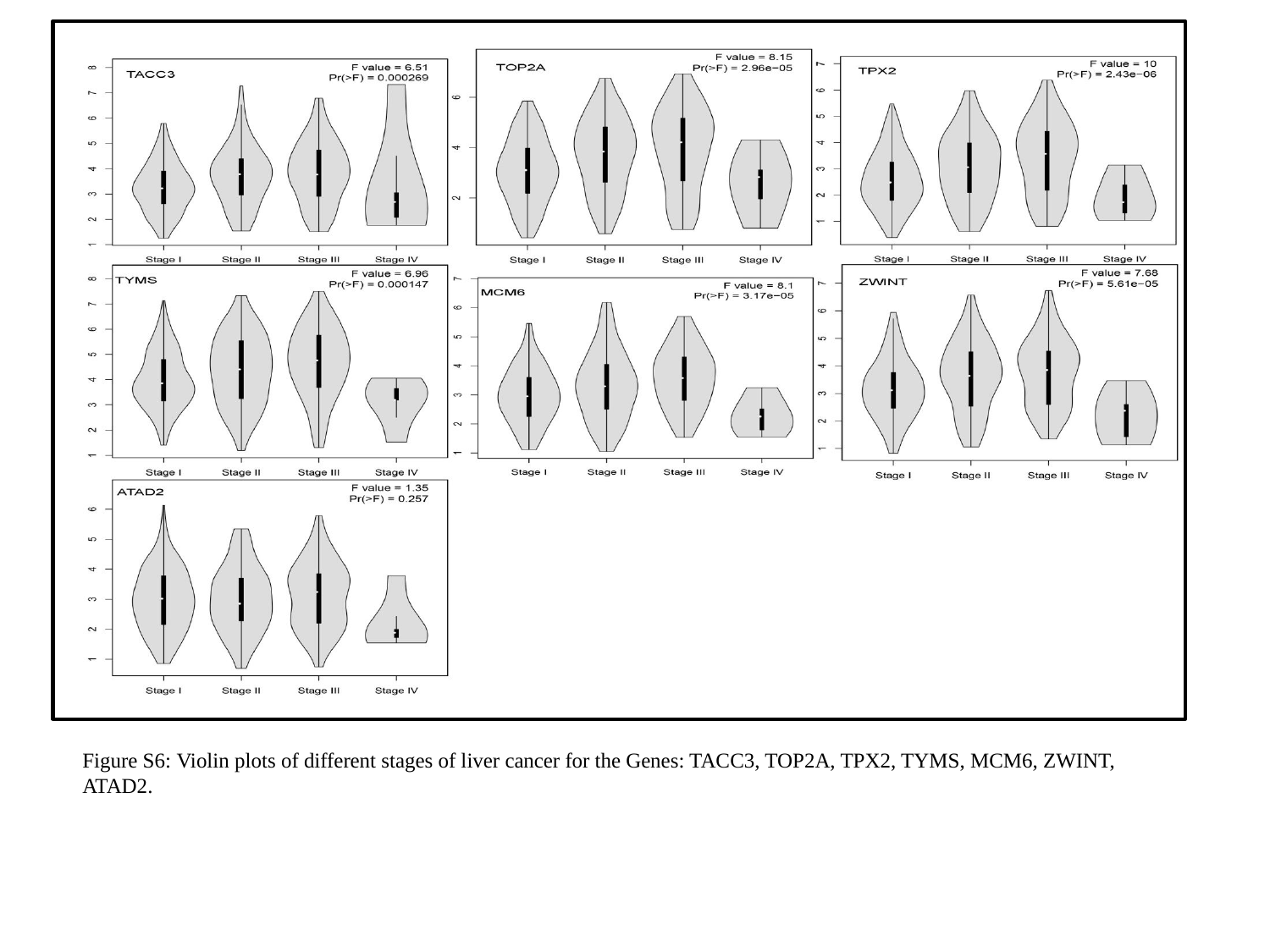

Figure S6: Violin plots of different stages of liver cancer for the Genes: TACC3, TOP2A, TPX2, TYMS, MCM6, ZWINT, ATAD2.

## Slide 8
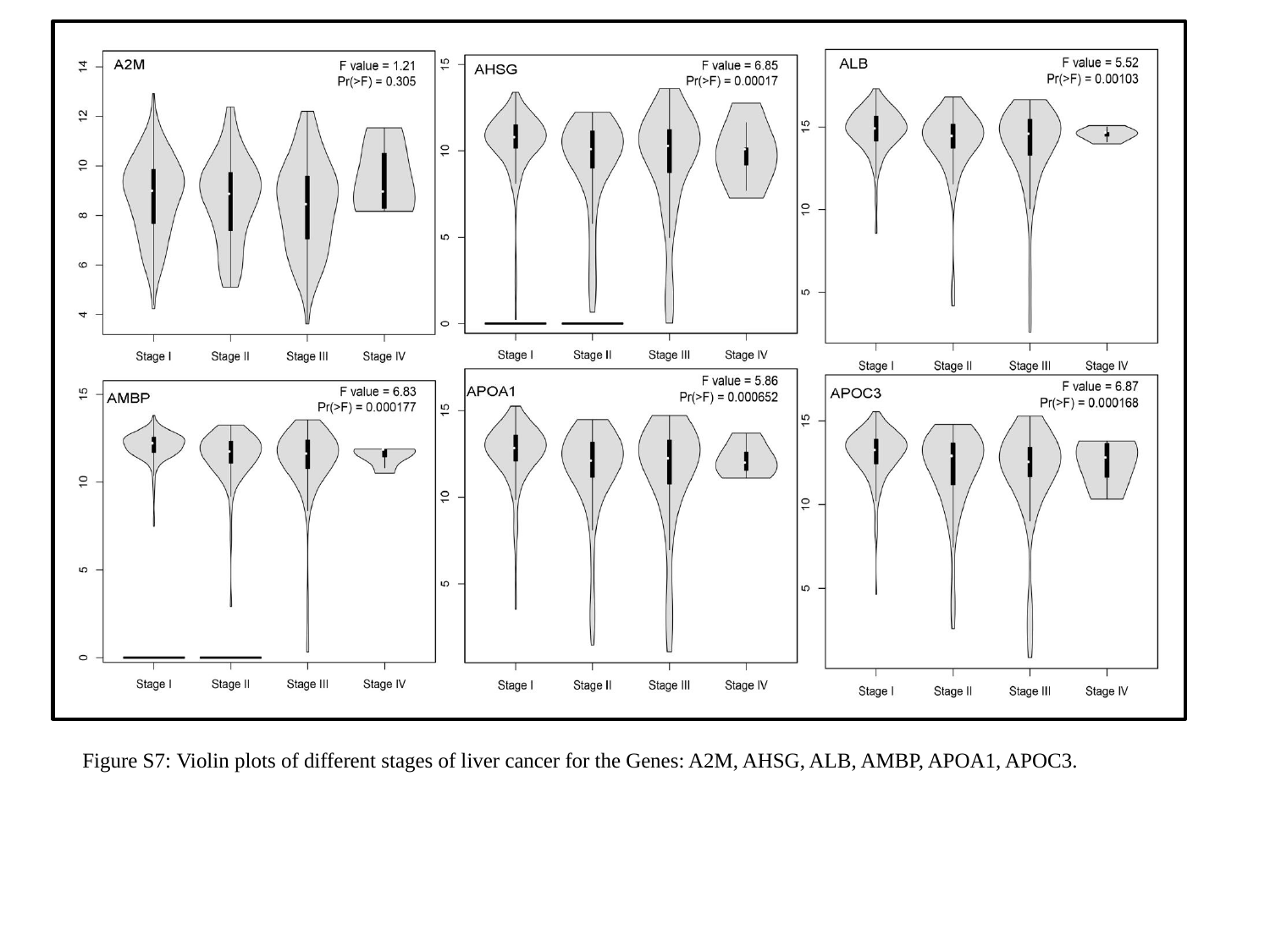

Figure S7: Violin plots of different stages of liver cancer for the Genes: A2M, AHSG, ALB, AMBP, APOA1, APOC3.

## Slide 9
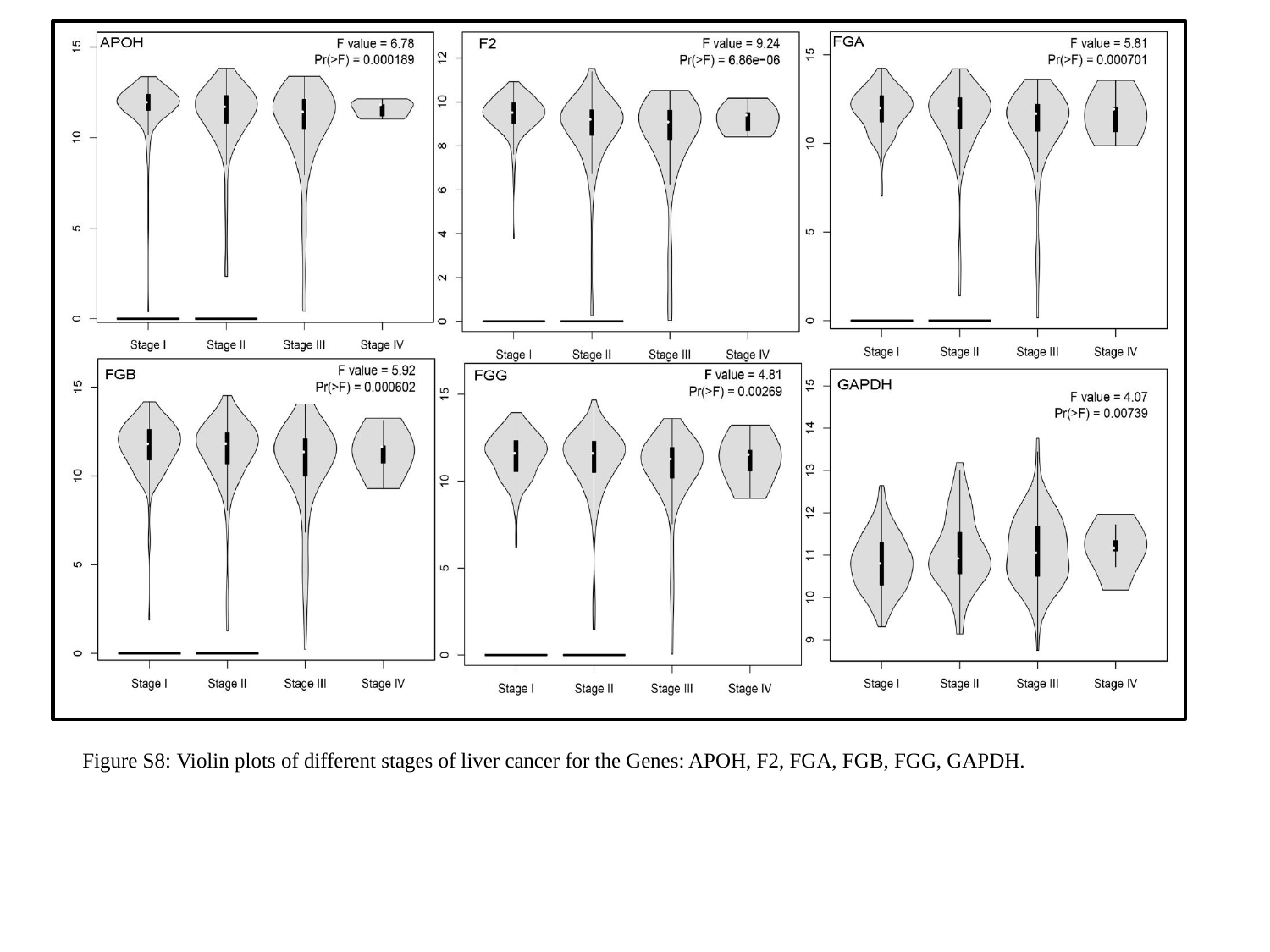

Figure S8: Violin plots of different stages of liver cancer for the Genes: APOH, F2, FGA, FGB, FGG, GAPDH.

## Slide 10
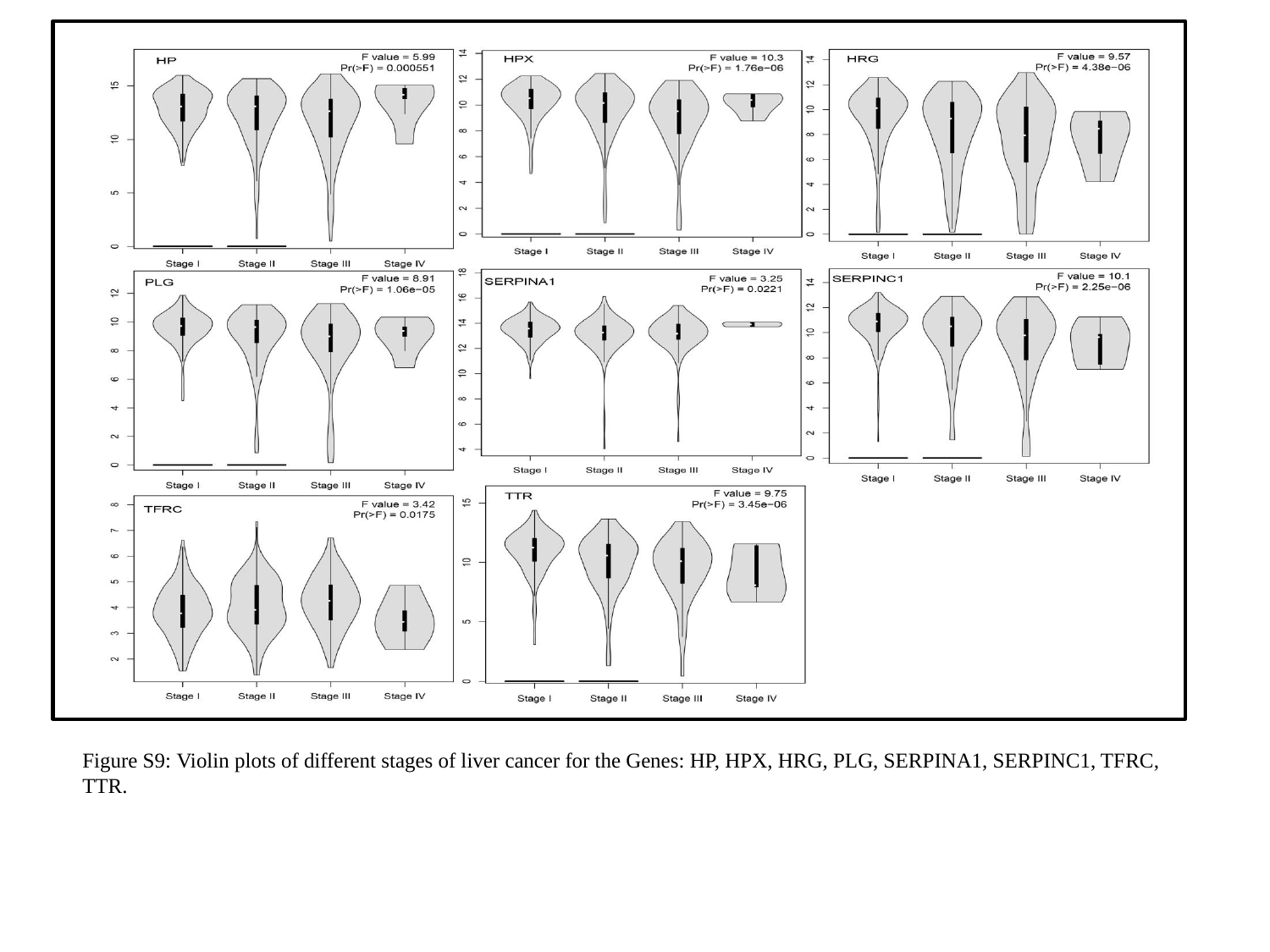

Figure S9: Violin plots of different stages of liver cancer for the Genes: HP, HPX, HRG, PLG, SERPINA1, SERPINC1, TFRC, TTR.

## Slide 11
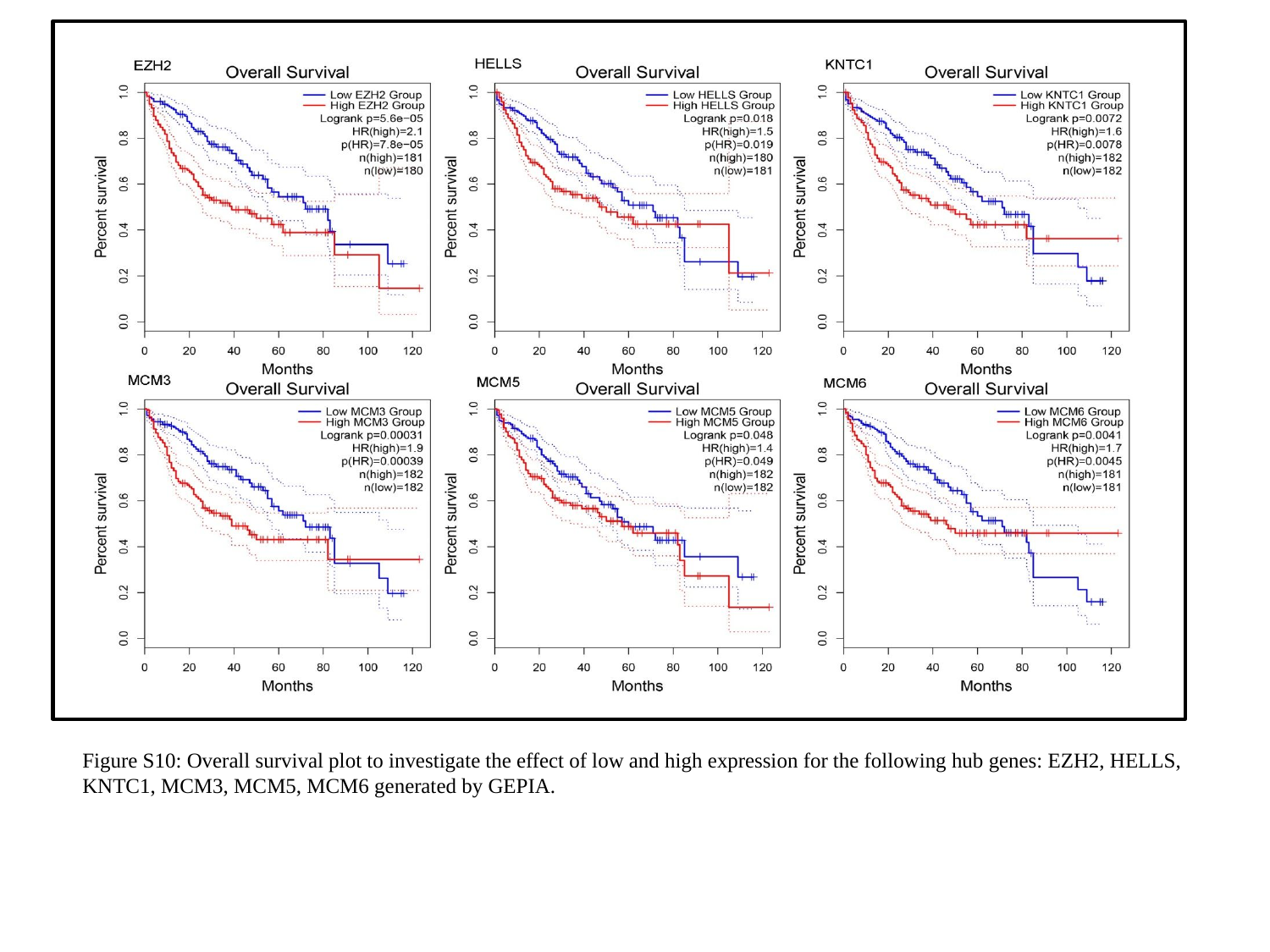

Figure S10: Overall survival plot to investigate the effect of low and high expression for the following hub genes: EZH2, HELLS, KNTC1, MCM3, MCM5, MCM6 generated by GEPIA.

## Slide 12
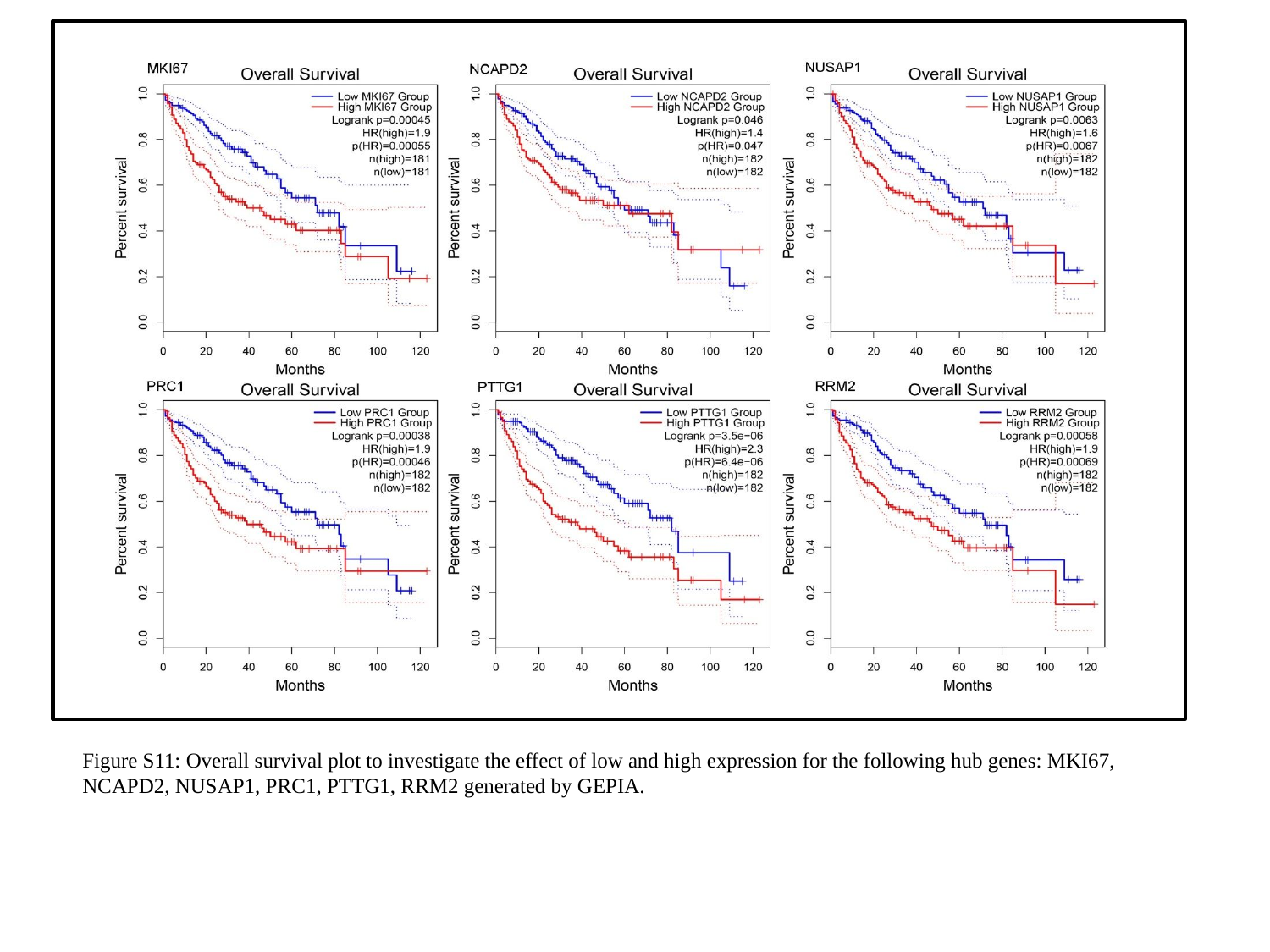

Figure S11: Overall survival plot to investigate the effect of low and high expression for the following hub genes: MKI67, NCAPD2, NUSAP1, PRC1, PTTG1, RRM2 generated by GEPIA.

## Slide 13
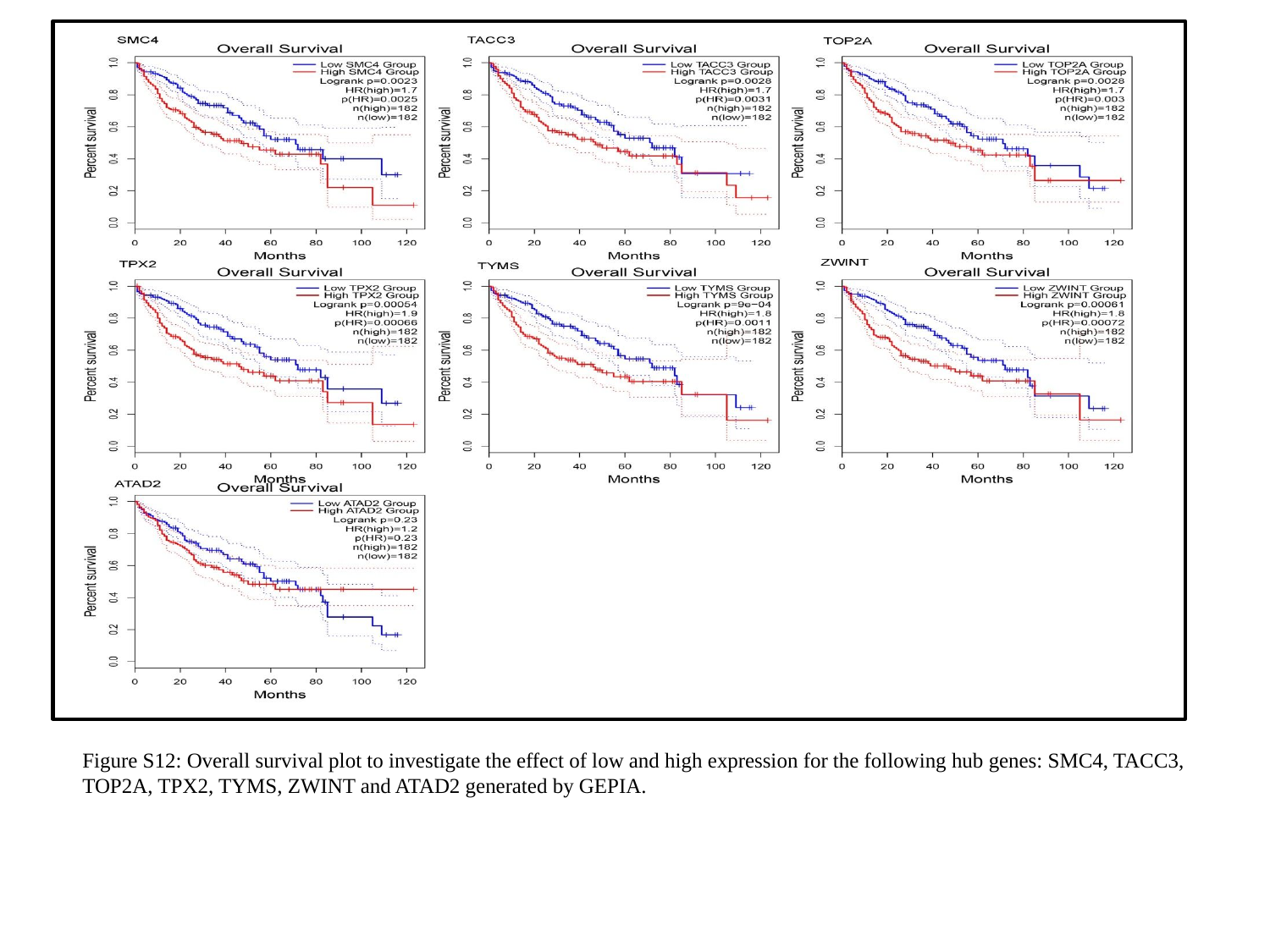

Figure S12: Overall survival plot to investigate the effect of low and high expression for the following hub genes: SMC4, TACC3, TOP2A, TPX2, TYMS, ZWINT and ATAD2 generated by GEPIA.

## Slide 14
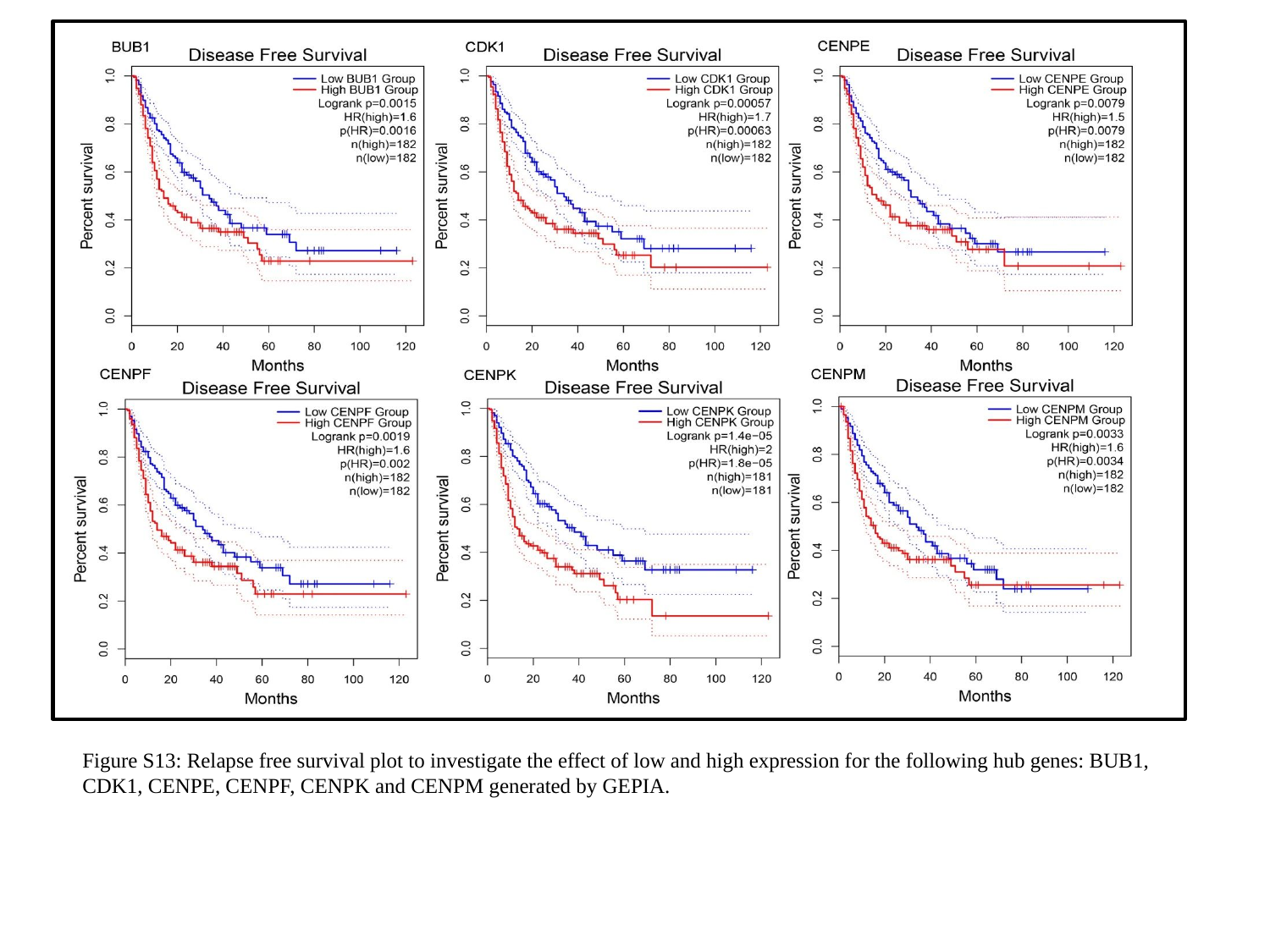

Figure S13: Relapse free survival plot to investigate the effect of low and high expression for the following hub genes: BUB1, CDK1, CENPE, CENPF, CENPK and CENPM generated by GEPIA.

## Slide 15
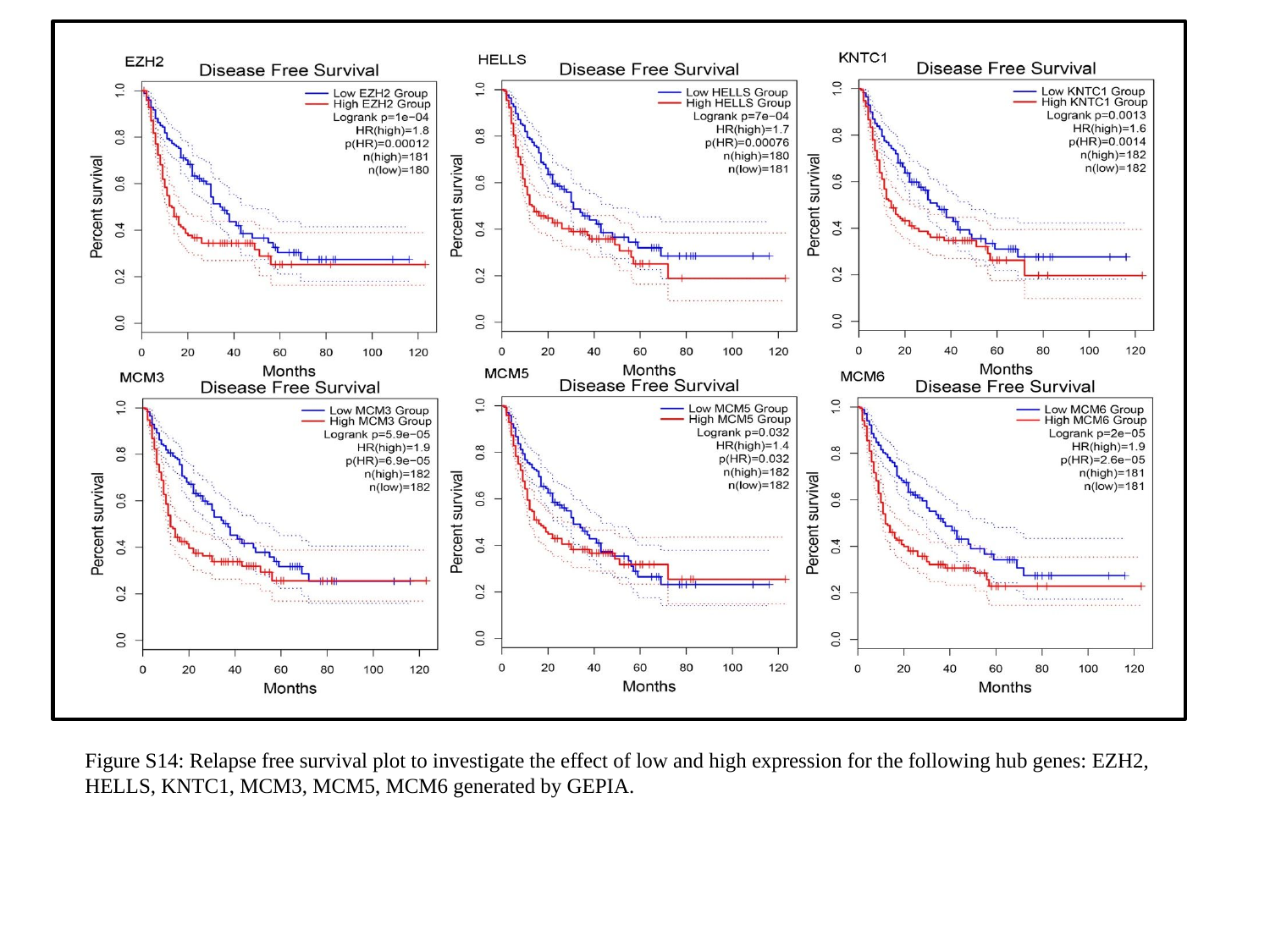

Figure S14: Relapse free survival plot to investigate the effect of low and high expression for the following hub genes: EZH2, HELLS, KNTC1, MCM3, MCM5, MCM6 generated by GEPIA.

## Slide 16
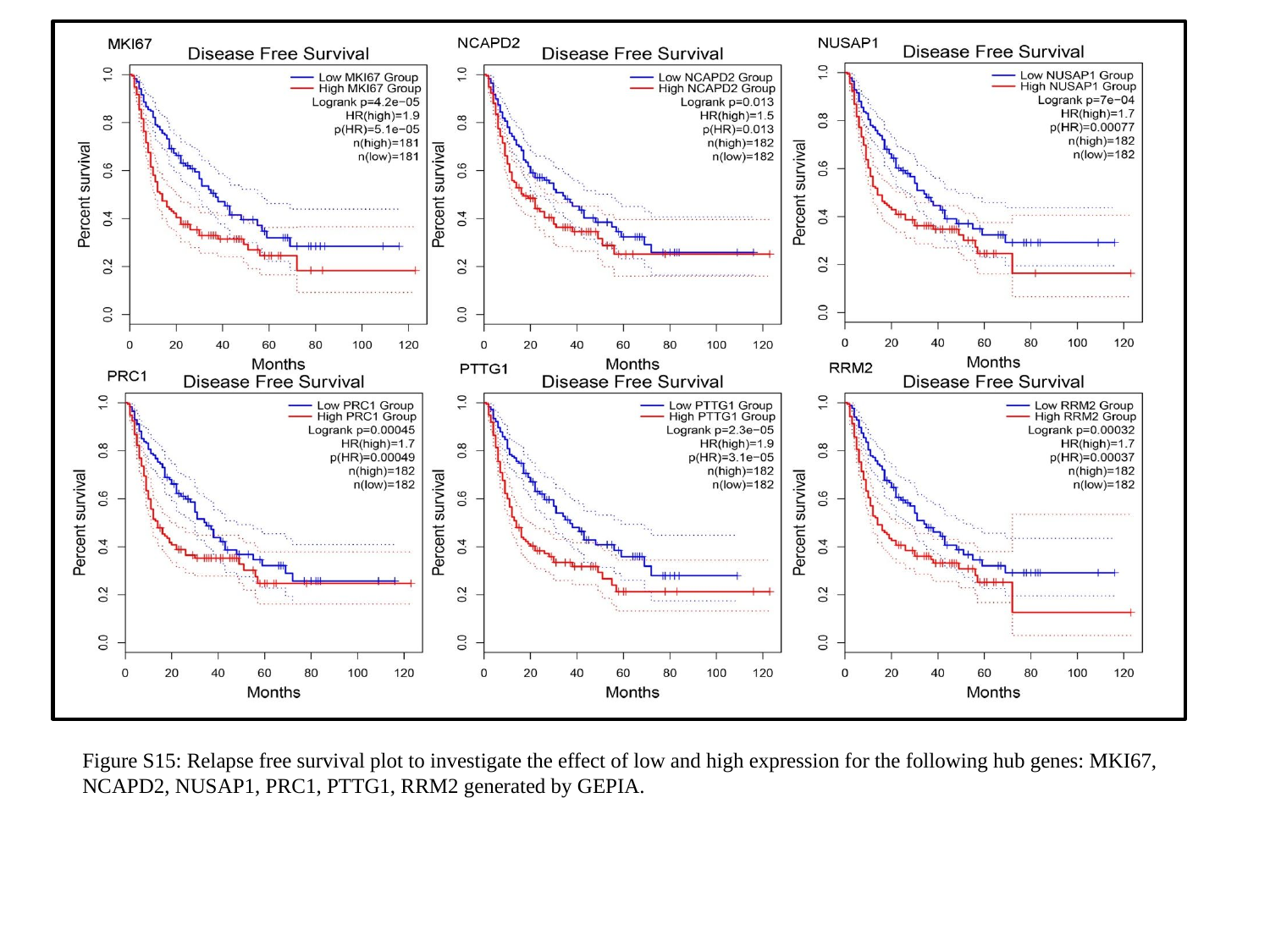

Figure S15: Relapse free survival plot to investigate the effect of low and high expression for the following hub genes: MKI67, NCAPD2, NUSAP1, PRC1, PTTG1, RRM2 generated by GEPIA.

## Slide 17
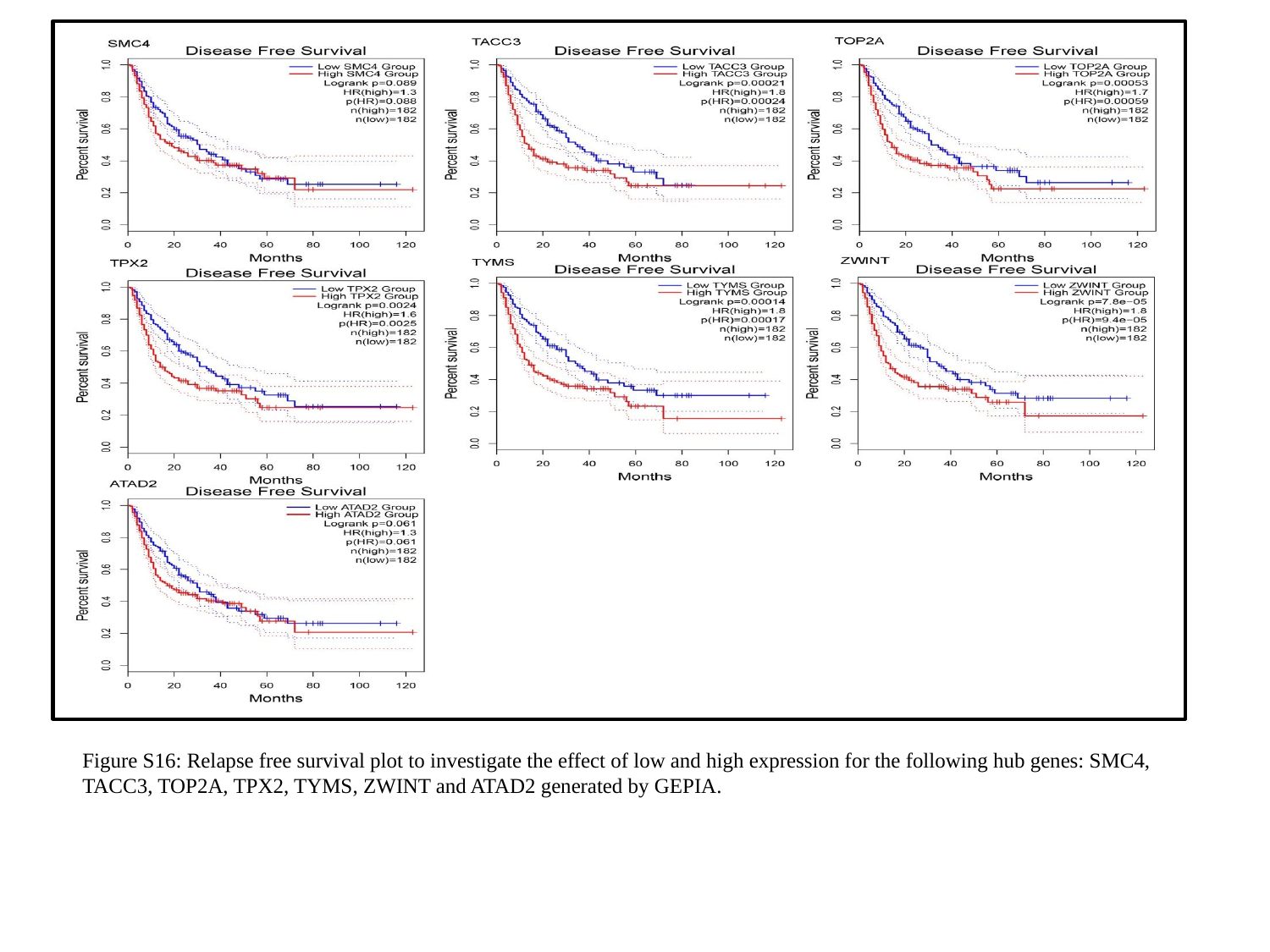

Figure S16: Relapse free survival plot to investigate the effect of low and high expression for the following hub genes: SMC4, TACC3, TOP2A, TPX2, TYMS, ZWINT and ATAD2 generated by GEPIA.

## Slide 18
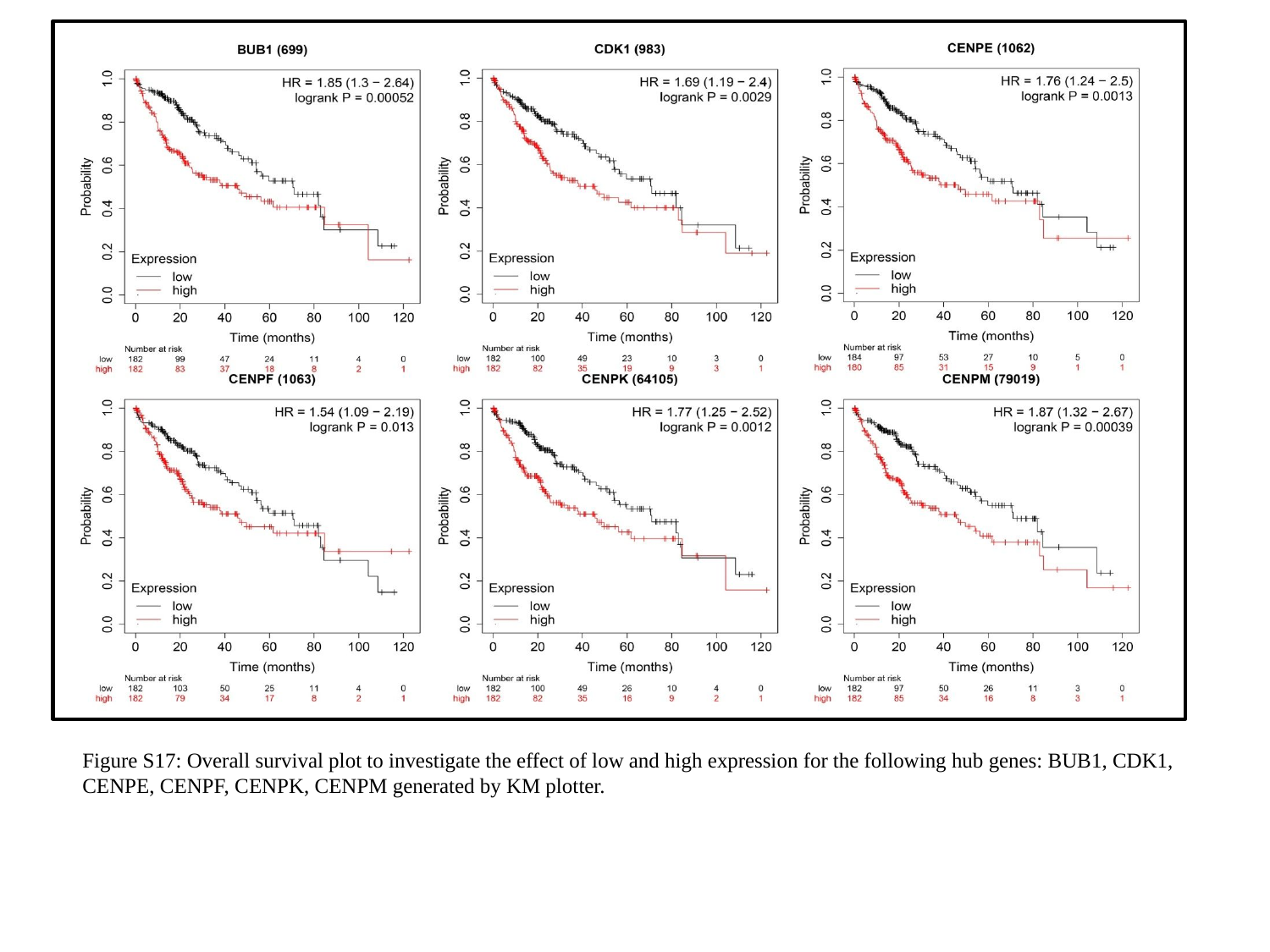

Figure S17: Overall survival plot to investigate the effect of low and high expression for the following hub genes: BUB1, CDK1, CENPE, CENPF, CENPK, CENPM generated by KM plotter.

## Slide 19
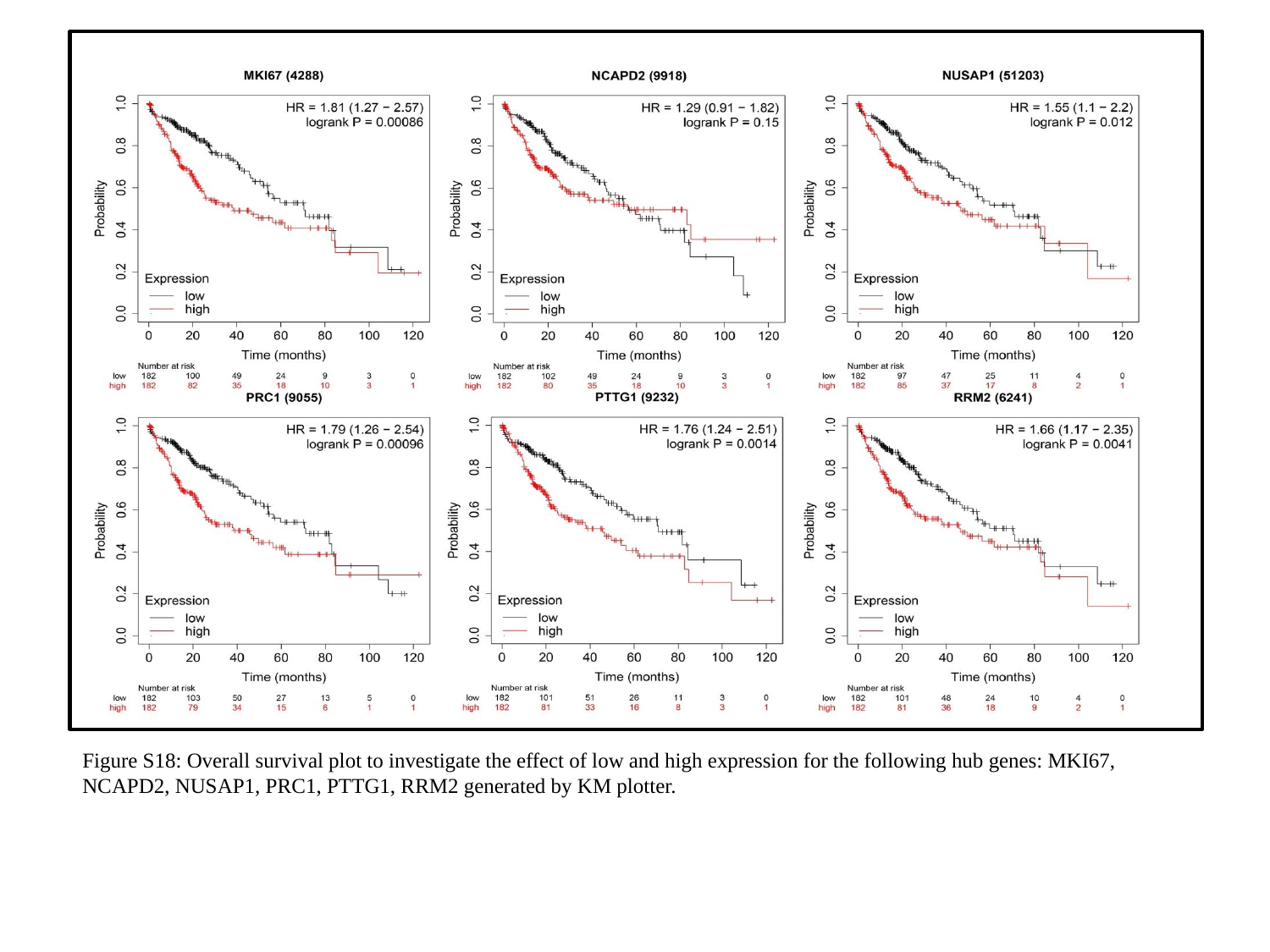

Figure S18: Overall survival plot to investigate the effect of low and high expression for the following hub genes: MKI67, NCAPD2, NUSAP1, PRC1, PTTG1, RRM2 generated by KM plotter.

## Slide 20
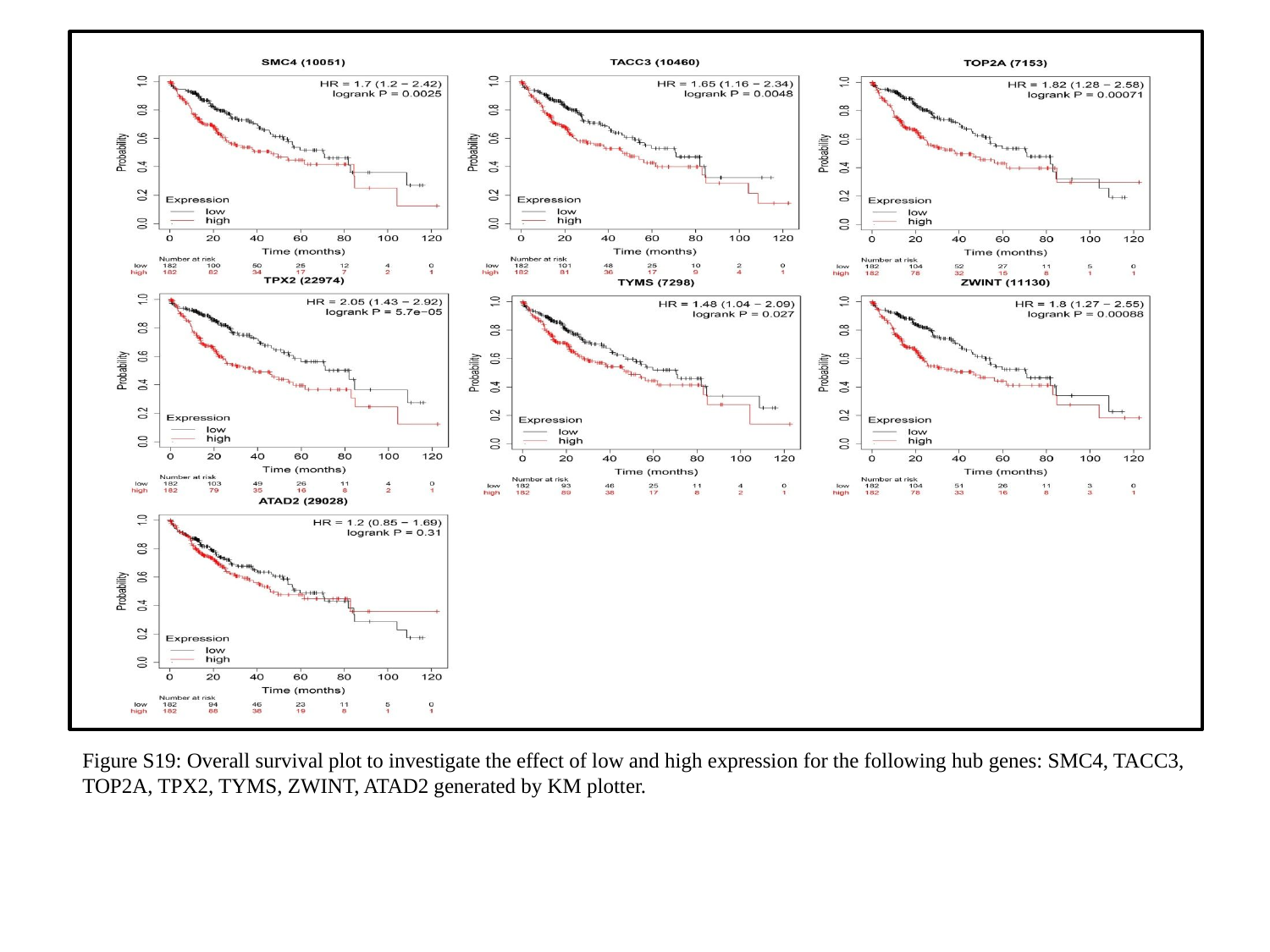

Figure S19: Overall survival plot to investigate the effect of low and high expression for the following hub genes: SMC4, TACC3, TOP2A, TPX2, TYMS, ZWINT, ATAD2 generated by KM plotter.

## Slide 21
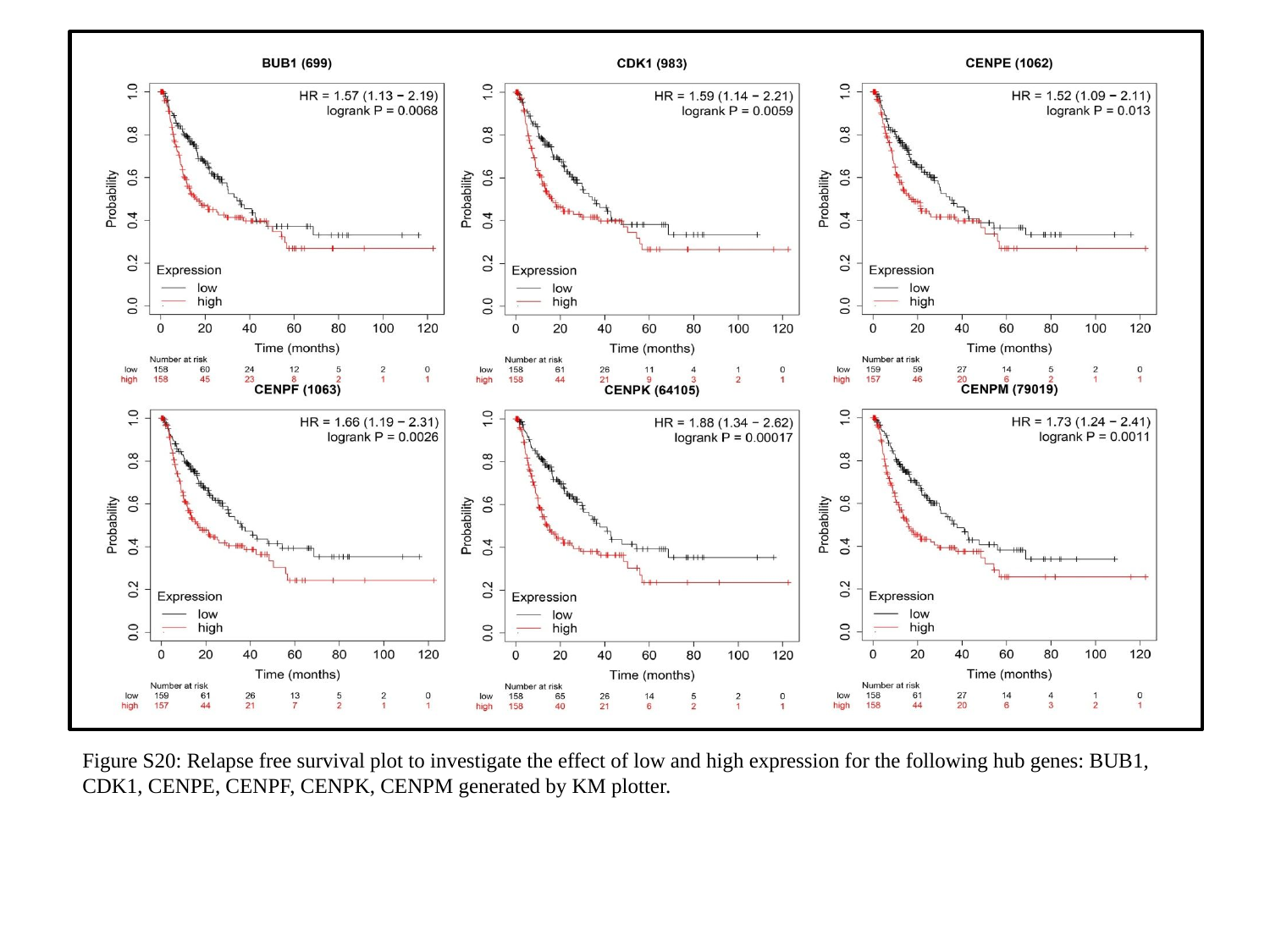

Figure S20: Relapse free survival plot to investigate the effect of low and high expression for the following hub genes: BUB1, CDK1, CENPE, CENPF, CENPK, CENPM generated by KM plotter.

## Slide 22
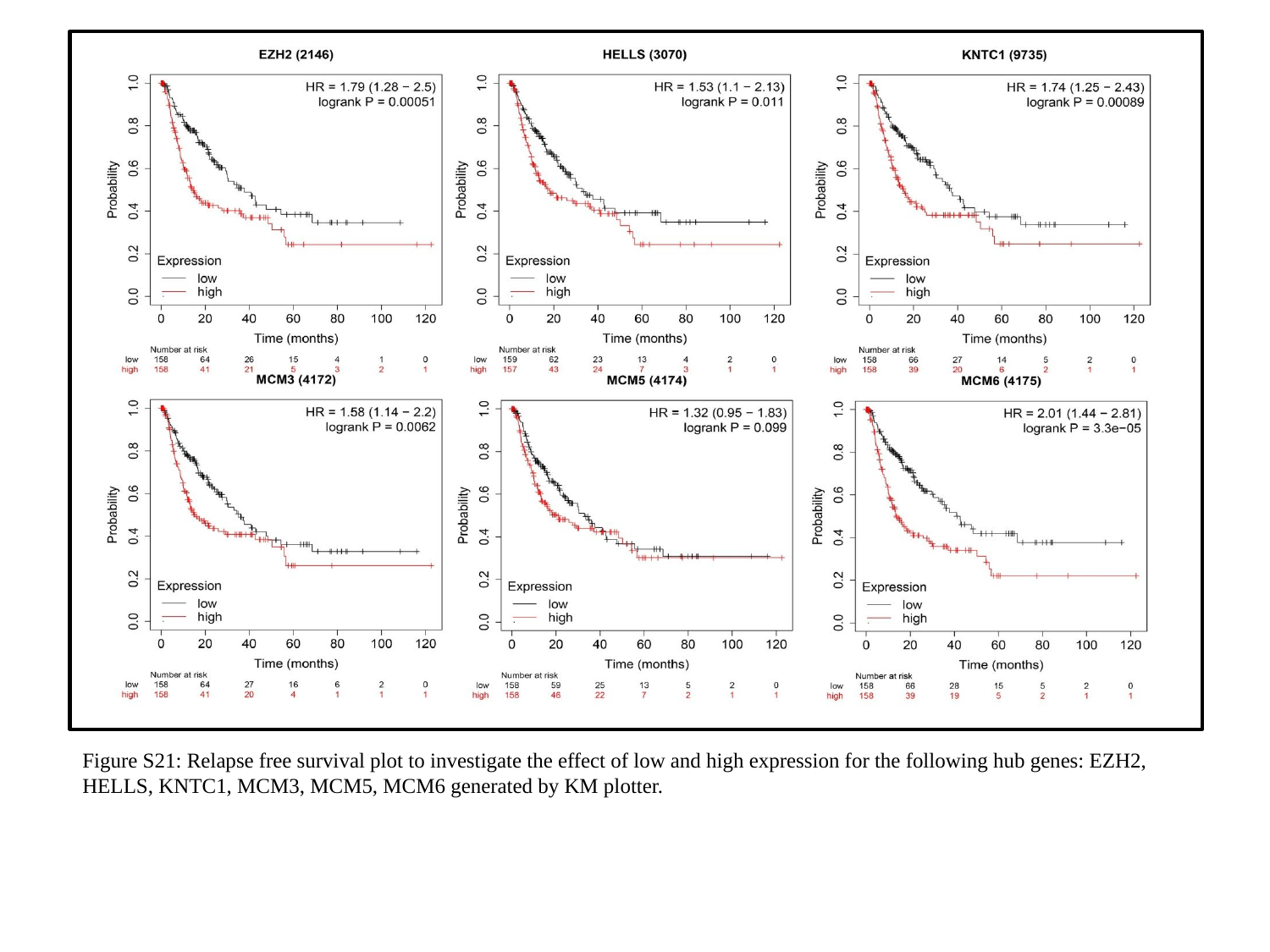

Figure S21: Relapse free survival plot to investigate the effect of low and high expression for the following hub genes: EZH2, HELLS, KNTC1, MCM3, MCM5, MCM6 generated by KM plotter.

## Slide 23
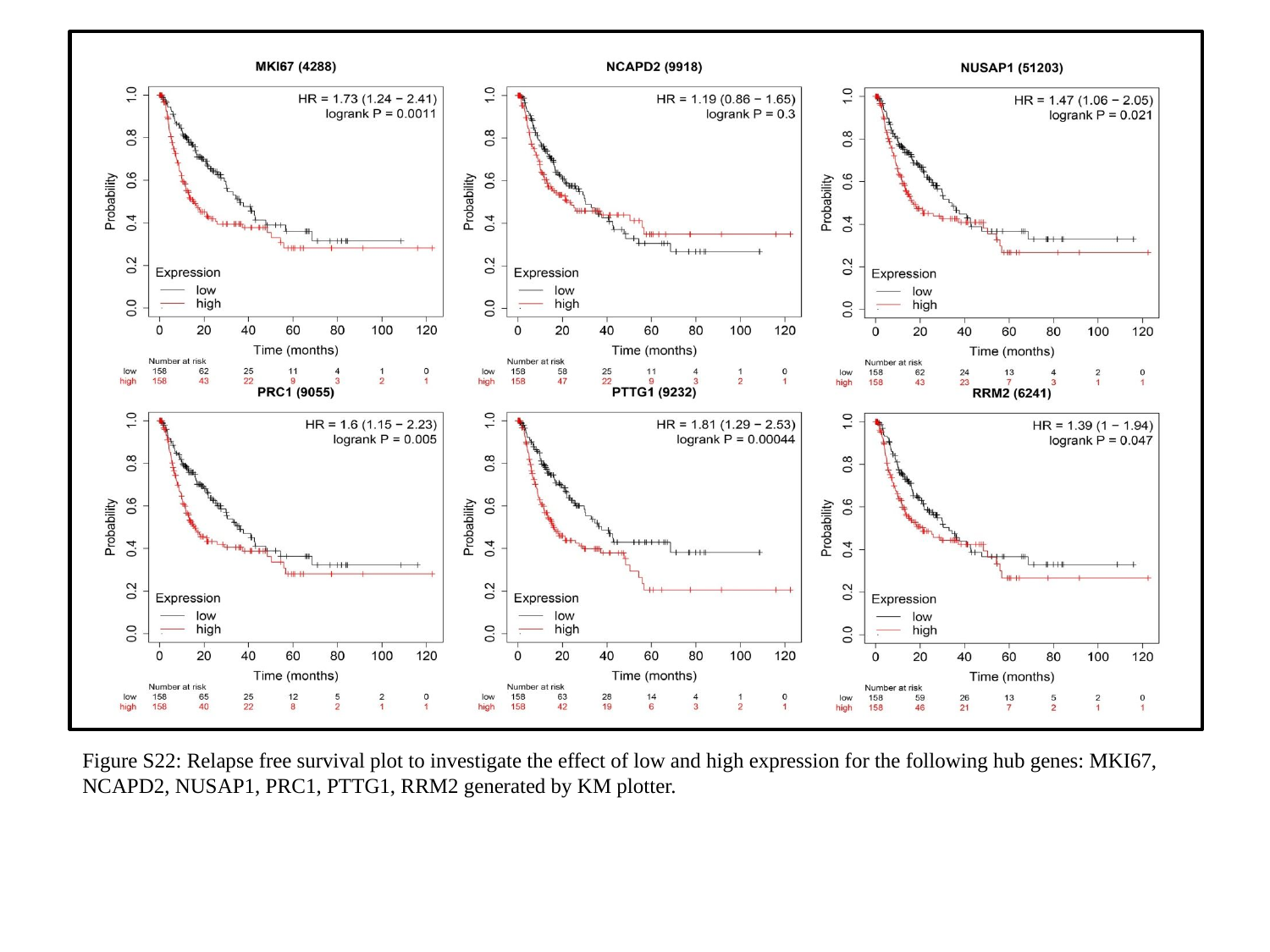

Figure S22: Relapse free survival plot to investigate the effect of low and high expression for the following hub genes: MKI67, NCAPD2, NUSAP1, PRC1, PTTG1, RRM2 generated by KM plotter.

## Slide 24
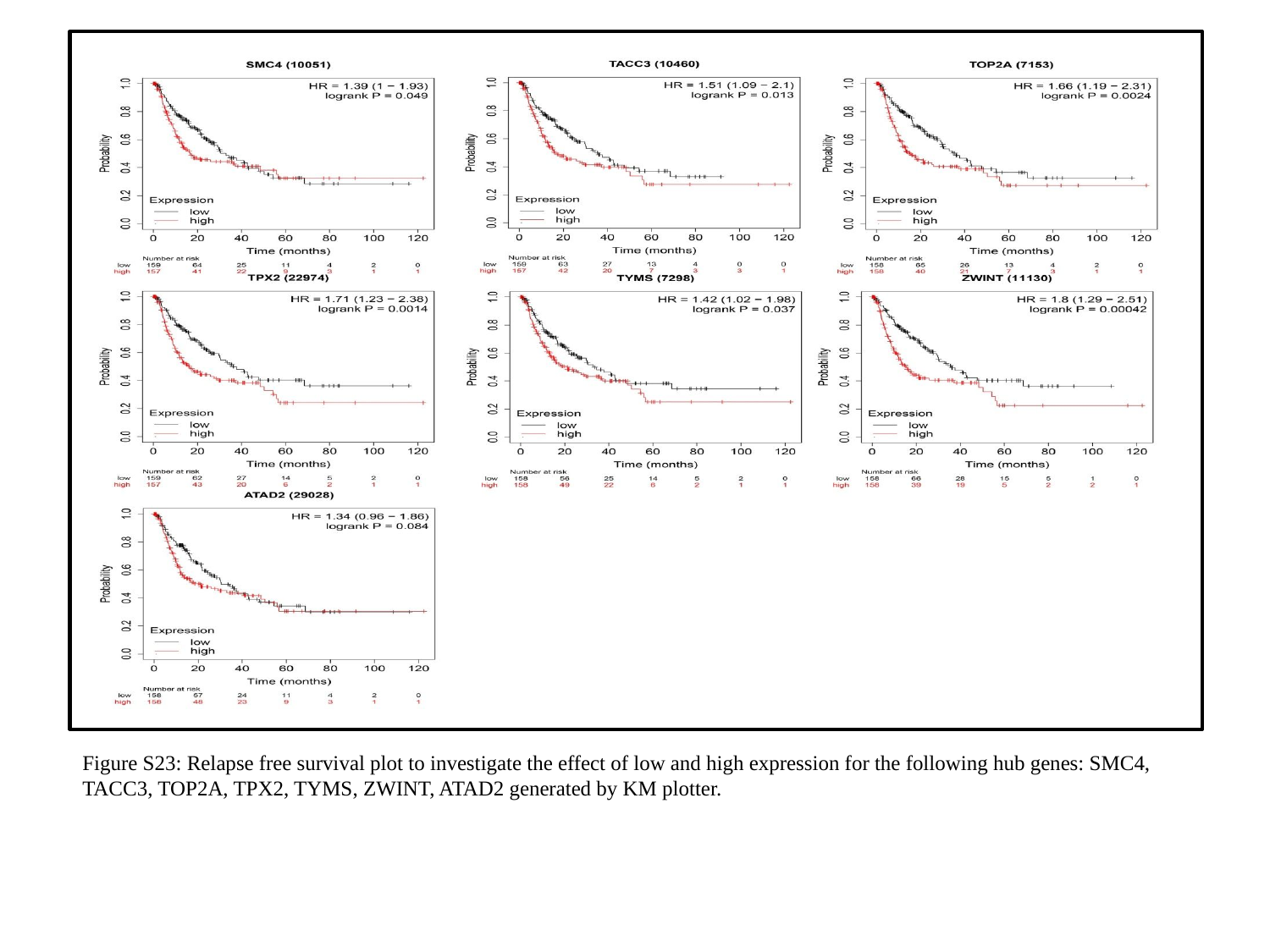

Figure S23: Relapse free survival plot to investigate the effect of low and high expression for the following hub genes: SMC4, TACC3, TOP2A, TPX2, TYMS, ZWINT, ATAD2 generated by KM plotter.

## Slide 25
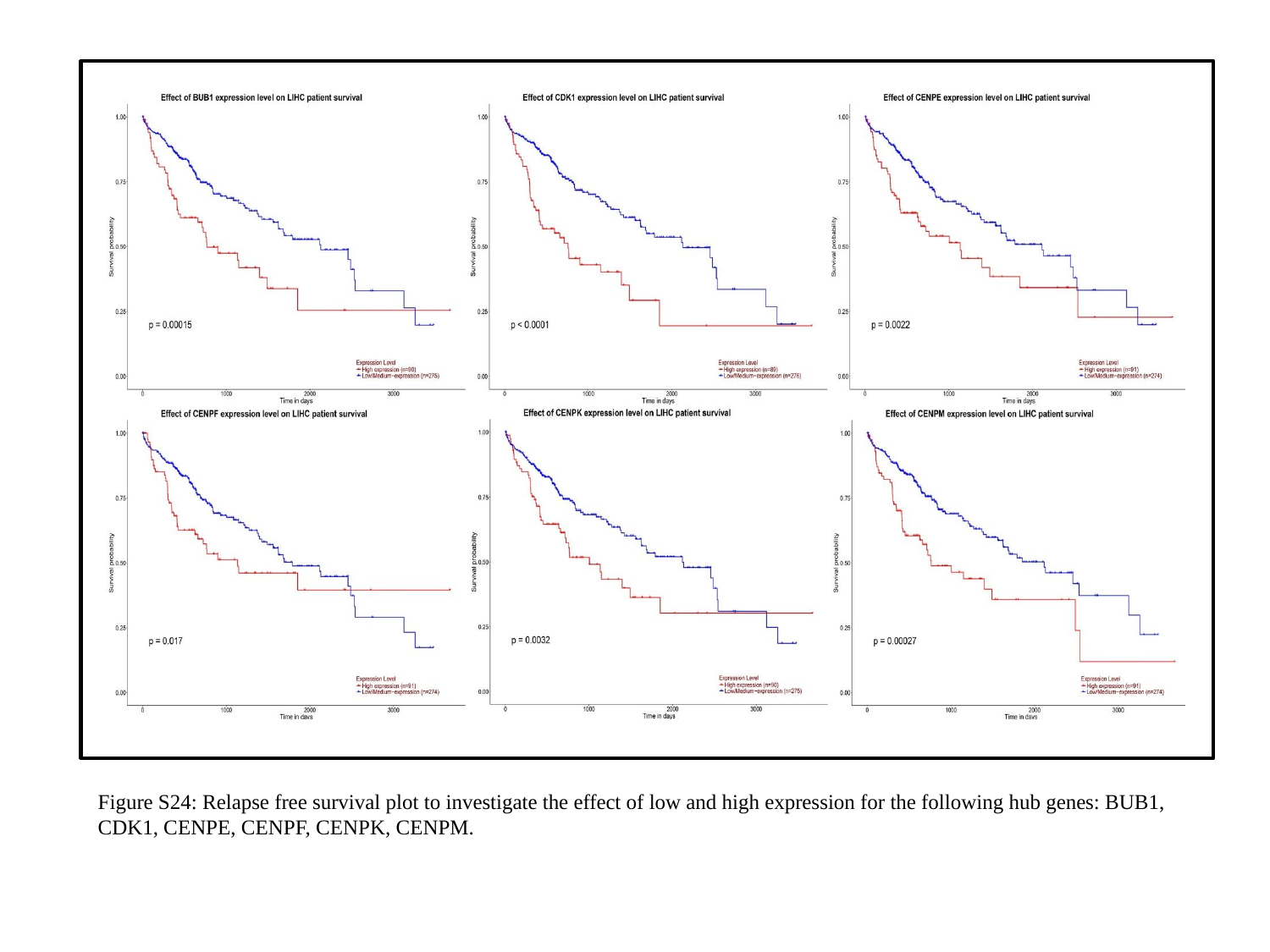

Figure S24: Relapse free survival plot to investigate the effect of low and high expression for the following hub genes: BUB1, CDK1, CENPE, CENPF, CENPK, CENPM.

## Slide 26
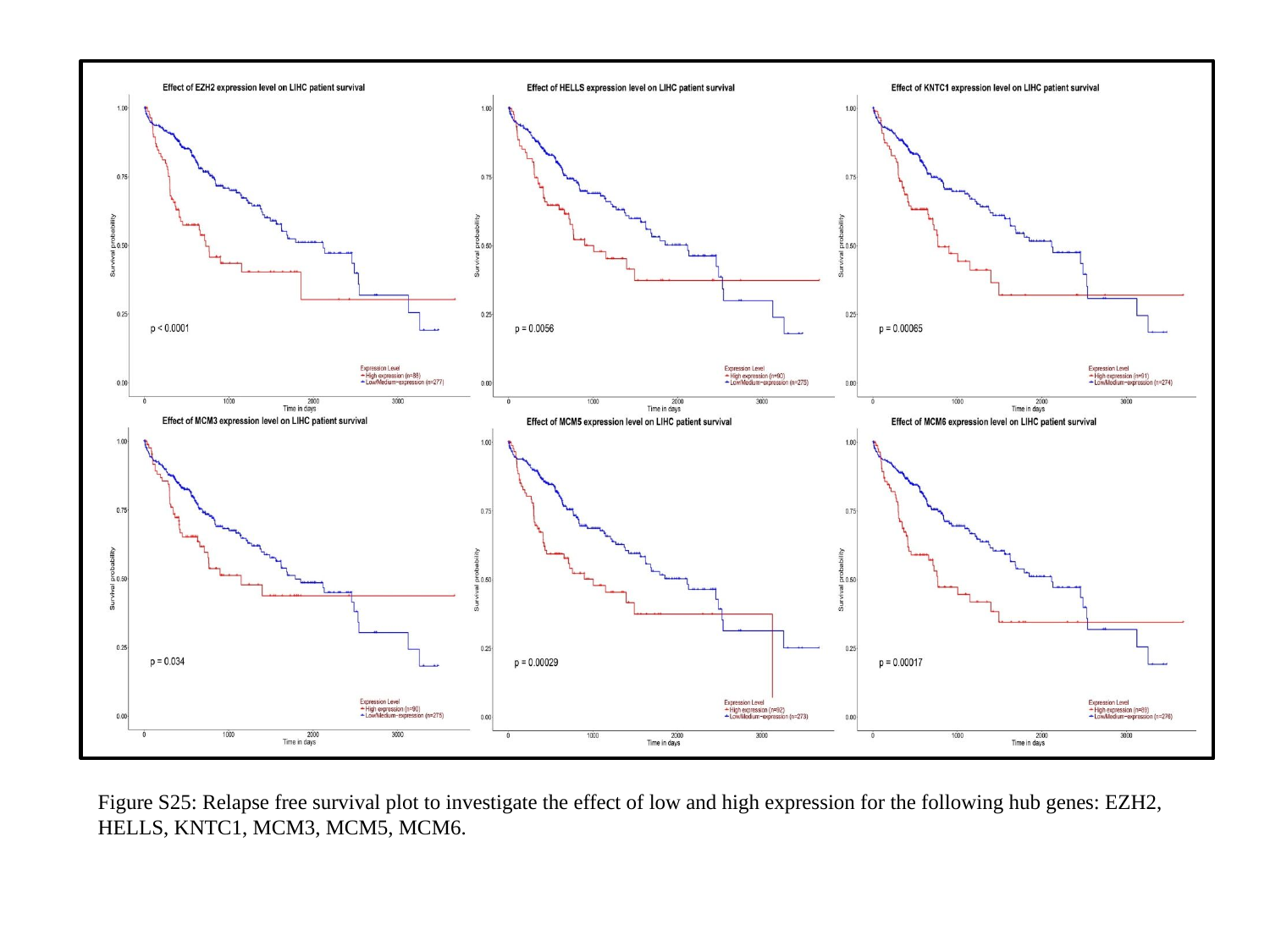

Figure S25: Relapse free survival plot to investigate the effect of low and high expression for the following hub genes: EZH2, HELLS, KNTC1, MCM3, MCM5, MCM6.

## Slide 27
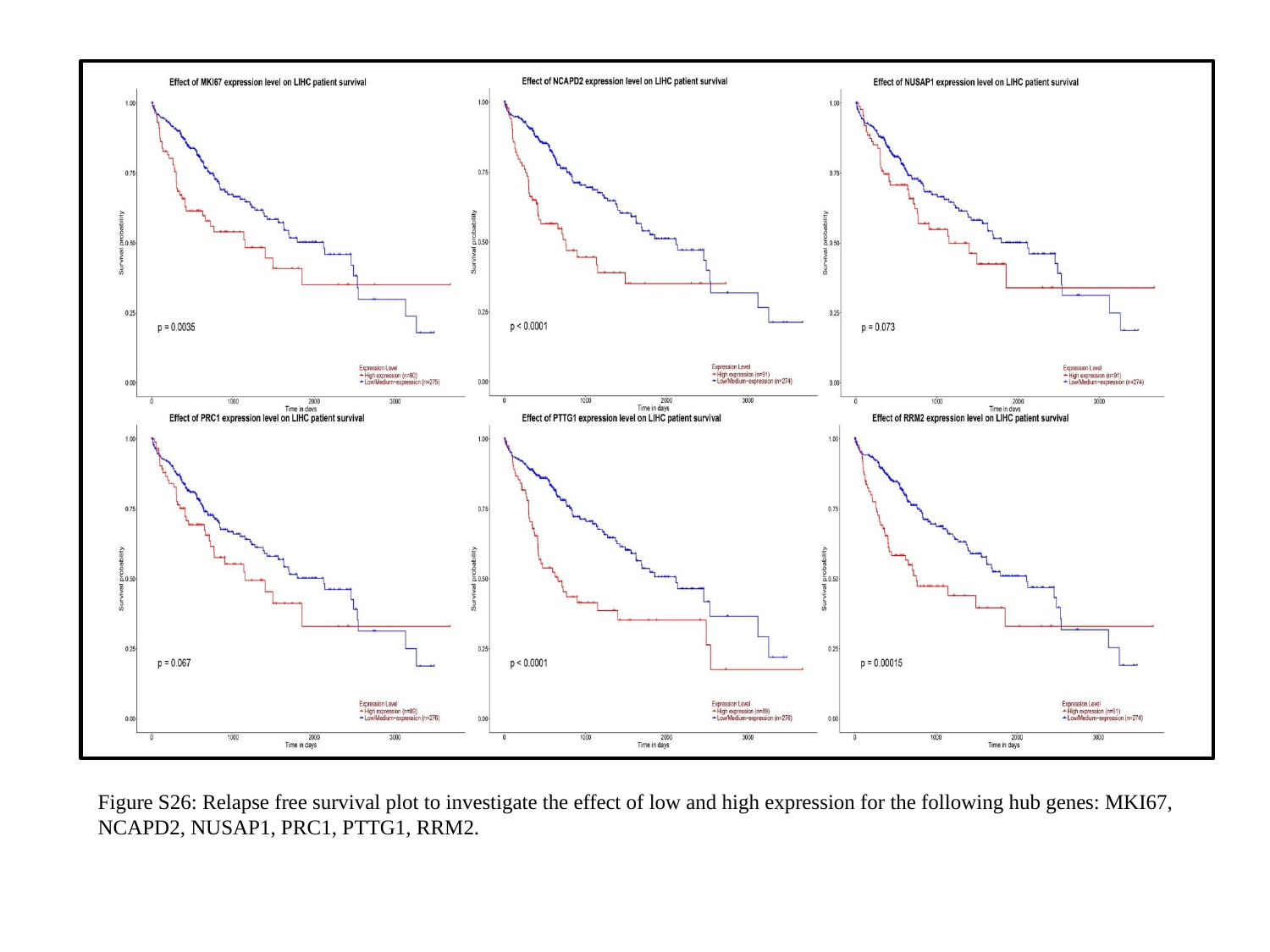

Figure S26: Relapse free survival plot to investigate the effect of low and high expression for the following hub genes: MKI67, NCAPD2, NUSAP1, PRC1, PTTG1, RRM2.

## Slide 28
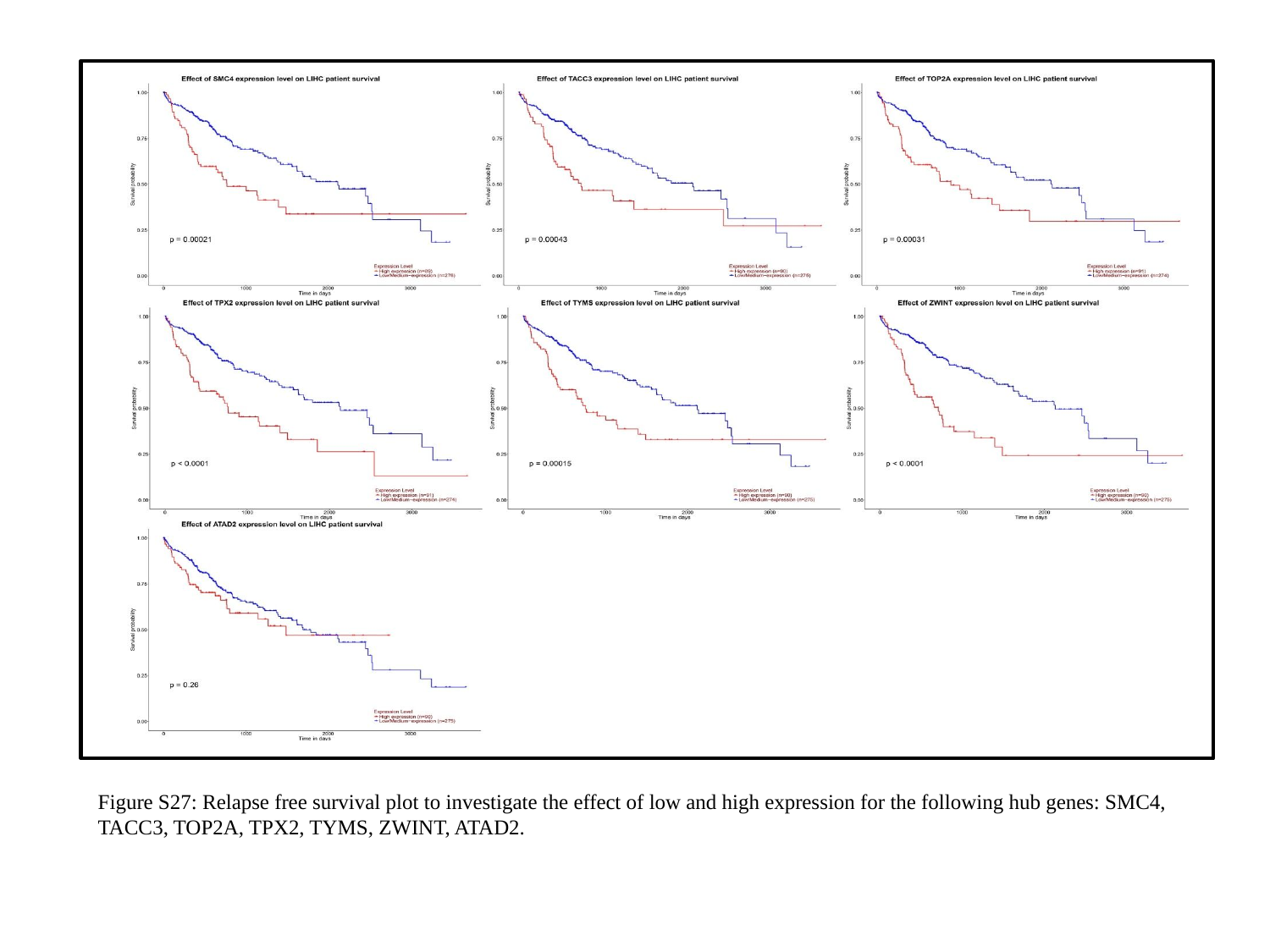

Figure S27: Relapse free survival plot to investigate the effect of low and high expression for the following hub genes: SMC4, TACC3, TOP2A, TPX2, TYMS, ZWINT, ATAD2.

## Slide 29
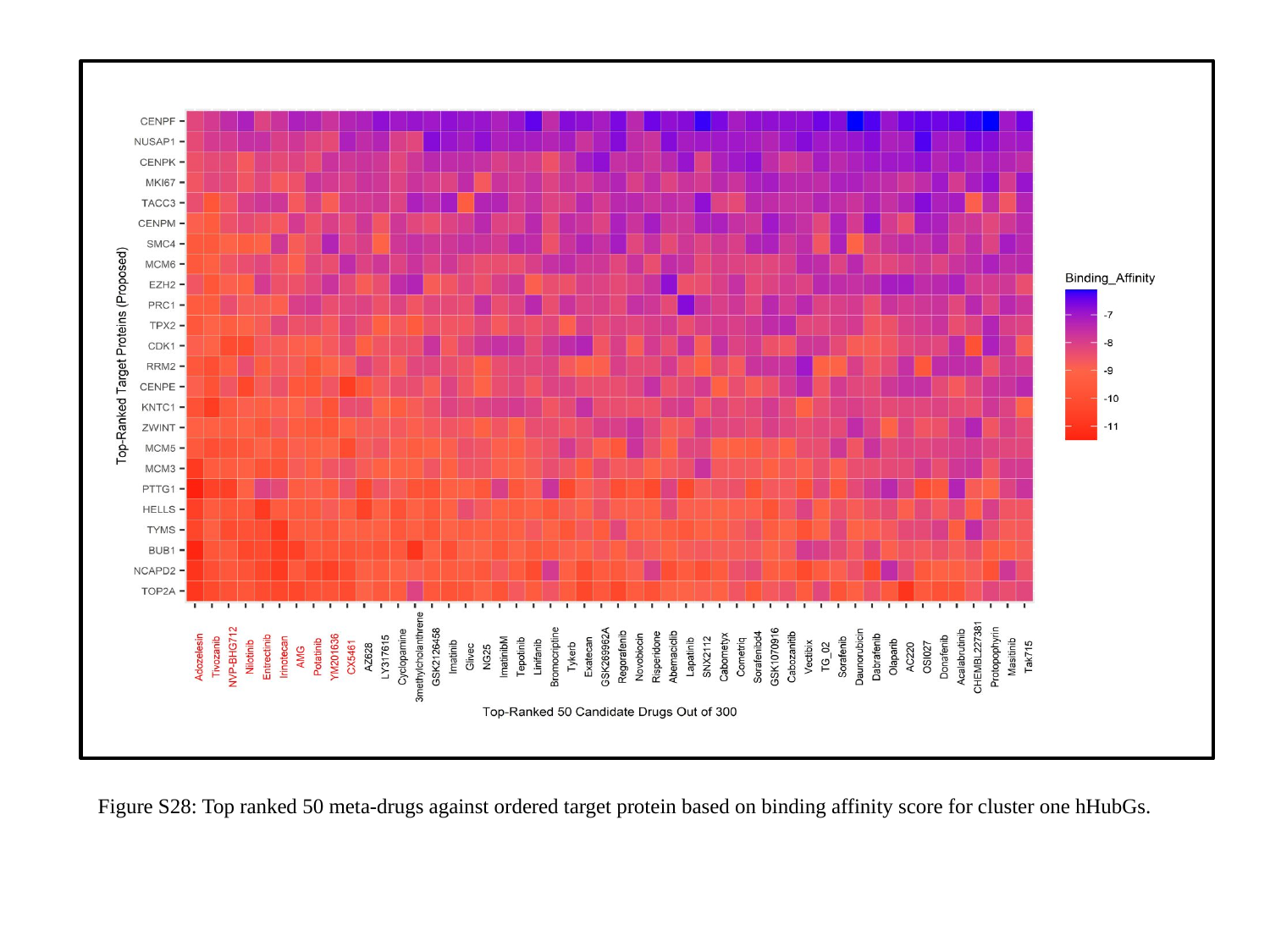

Figure S28: Top ranked 50 meta-drugs against ordered target protein based on binding affinity score for cluster one hHubGs.

## Slide 30
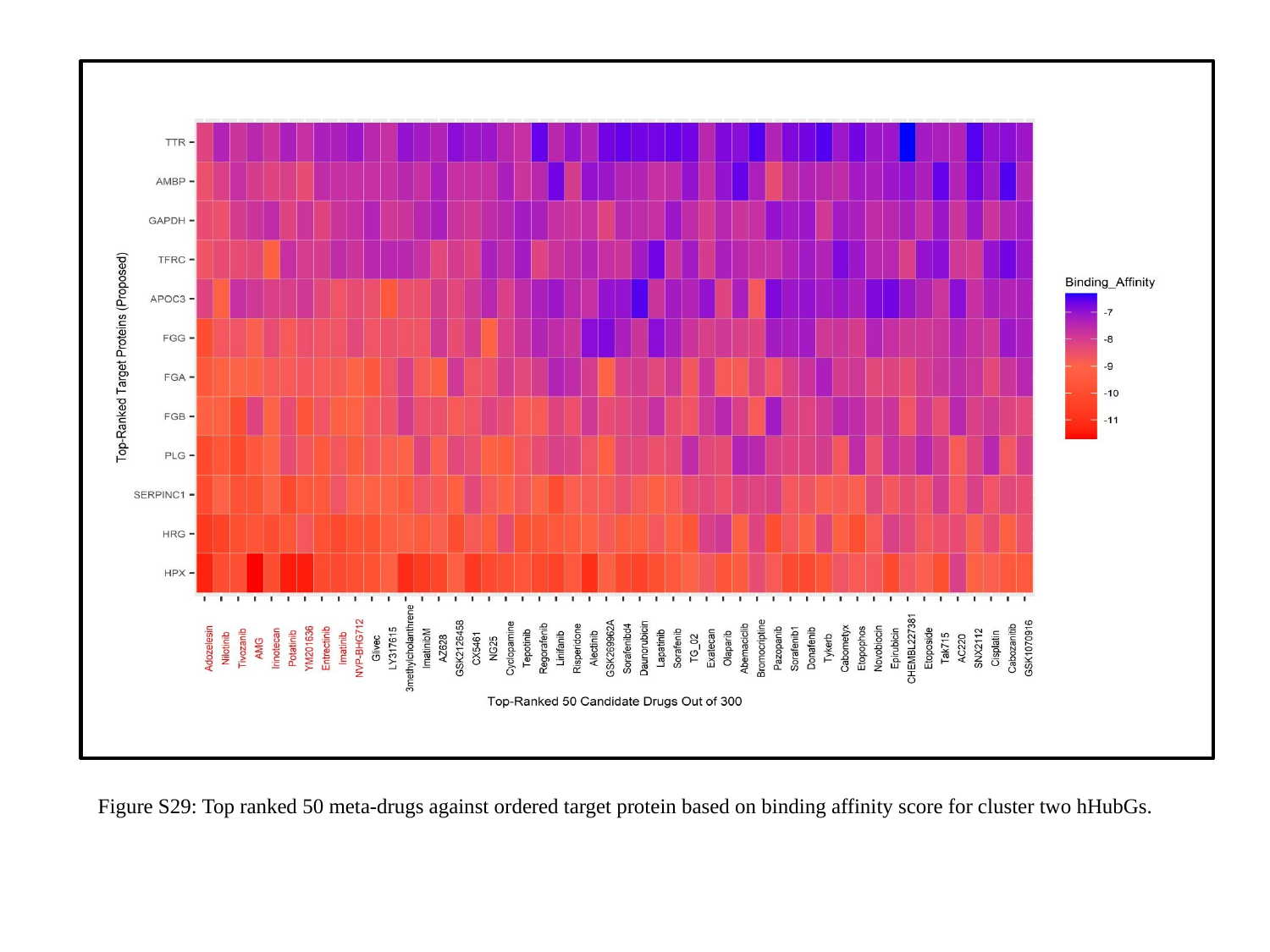

Figure S29: Top ranked 50 meta-drugs against ordered target protein based on binding affinity score for cluster two hHubGs.

## Slide 31
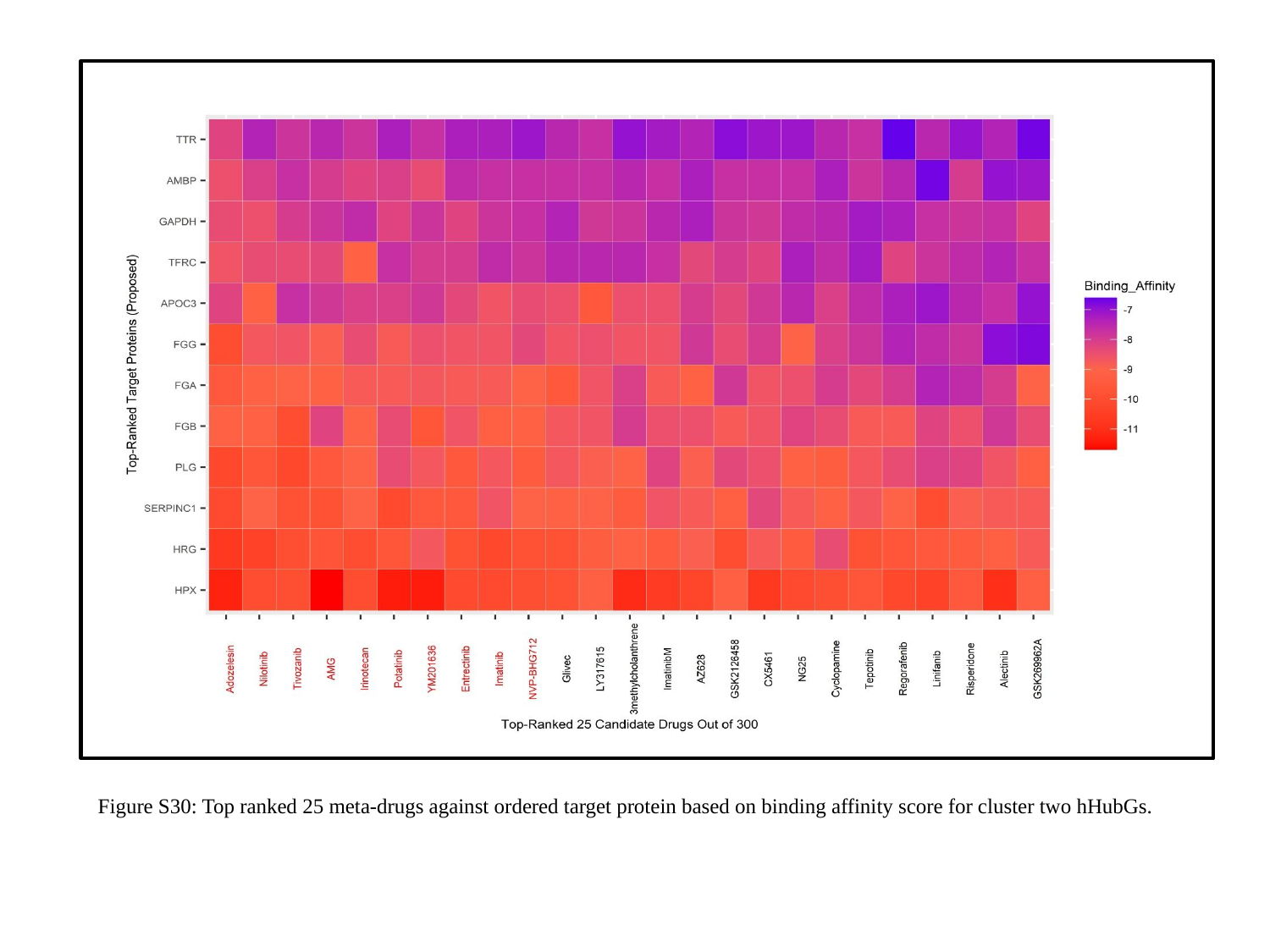

Figure S30: Top ranked 25 meta-drugs against ordered target protein based on binding affinity score for cluster two hHubGs.

## Slide 32
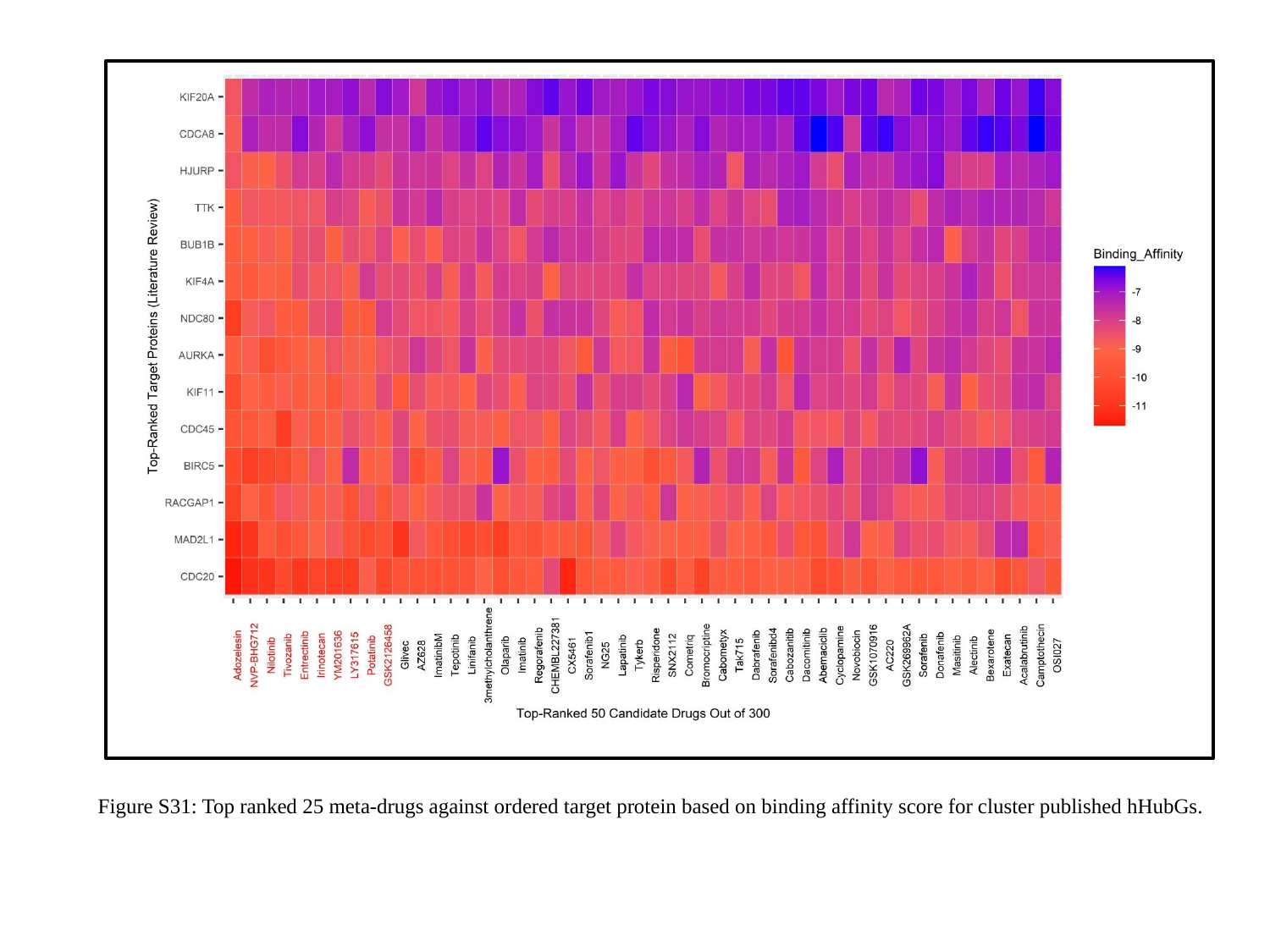

Figure S31: Top ranked 25 meta-drugs against ordered target protein based on binding affinity score for cluster published hHubGs.
