# Supplementary material for: Discovery of Genomic Targets and Therapeutic Candidates for Liver Cancer Using Single-Cell RNA Sequencing and Molecular Docking
Source: Biology (Basel). 2025 Apr 17;14(4):431. doi: 10.3390/biology14040431 (PMC12024973; doi:10.3390/biology14040431)
Supplement: Supplementary file 1 [file biology-14-00431-s001.zip › Supplementary Table.pdf]

Supplementary Table On "**Discovery of Genomic Targets and Therapeutic Candidates for Liver Cancer Using Single-Cell RNA Sequencing and Molecular Docking**"

Biplab Biswas[a,b], Masahiro Sugimoto[c,d], Md. Aminul Hoque[b*]

[a] Department of Statistics, Faculty of Science, Gopalganj Science & Technology University, Gopalganj-8100, Bangladesh,

[b] Department of Statistics, Faculty of Science, University of Rajshahi, Rajshahi-6205, Bangladesh.

[c] Institute for Advanced Biosciences, Keio University, Tsuruoka, Yamagata, Japan.

[d] Institute of Medical Science, Tokyo Medical University, Tokyo, Japan.

Table S1: Top common gene ontology (Molecular function and Cellular component) from four databases.

Category	GO ID	Terms	Associated Gene Count				Common Associated Genes
			DAVID	String	Enrichr	WebGestalt	
Molecular Function	GO:0005515	protein binding	44	34	-	-	ATAD2, TOP2A, PTTG1, TPX2, ZWINT, ALB, A2M, APOA1, APOC3, APOH, CENPE, CENPF, F2, CDK1, EZH2, FGA,FGB, FGG, GAPDH, HP, HRG, KNTC1, MCM6, NCAPD2,NUSAP1, PLG, PRC1, RRM2, SERPINA1, SERPINC1, TFRC, TTR
	GO:0042802	identical protein binding	12	18	-	14	ALB, A2M, APOA1, APOH, FGG, GADPH, MCM6, PRC1, SERPINA1,SERPINC1, TTR, CENPF,TOP2A, TYMS, TFRC, AMBP
	GO:0004866	endopeptidase inhibitor activity	02	08	06	08	AHSG, A2M, HRG, SERPIC1, AMBP, SERPINA1, GAPDH, PTTG1
	GO:0004867	serine-type endopeptidase inhibitor activity	04	05	03	05	AMBP, A2M, HRG, SERPINC1, SERPINA1
	GO:0003697	single-stranded DNA binding	04	04	03	-	MCM3, MCM5,MCM6,SMC4
	GO:0005524	ATP binding	11	-	02	12	ATAD2, BUB1, TOP2A, CENPE, CDK1, HELLS, MKI67, MCM3, MCM5, MCM6, SMC4
	GO:0042803	protein homodimerization activity	07	-	05	-	TOP2A, AMBP, APOA1, CENPF, TFRC
Cellular Component	GO:0004857	enzyme inhibitor activity	-	08	-	10	GAPDH, SERPINA1, APOC3, AMBP, SERPINC1, A2M, HRG, AHSG
	GO:0072562	blood microparticle	15	15	-	15	ALB, AHSG, AMBP, A2M, APOA1, F2, FGA, FGB, FGG, HP, HPX, HRG, PLG, SERPINC1, TFRC
	GO:0031093	platelet alpha granule lumen	09	09	08	09	ALB, AHSG, A2M, FGA, FGB,FGG, HRG, PLG, SERPINA1
	GO:0070062	Extracellular exosome	21	22	-	-	ATAD2, ALB, AHSG, AMBP, A2M, APOA1, APOC3, APOH, F2, CDK1, FGA,FGB,FGG, GAPDH,HP, HRG, PLG, SERPINA1, SERPINC1, TTR
	GO:0000779	Condensed Chromosome, Centromeric Region	06	08	02	-	CENPE, CENPF, CENPK, NCAPD2
	GO:0005788	Endoplasmic Reticulum Lumen	08	08	08	-	ALB, AHSG, APOA1, F2, FGA,FGG, SERPINA1, SERPINC1,
	GO:0005694	Chromosome	07	19	04	-	CENPE, MKI67, MCM3, NCAPD2, NUSAP1, PRC1, SMC4, TOP2A,
	GO:0062023	Collagen-Containing Extracellular Matrix	-	15	14	15	HRG, PLG, FGA,FGB, FGG, AHSG, A2M, APOH,SERPINC1, F2, AMBP, SERPINA1, SERPINC1, APOC3, HPX, APOA1
	GO:0098687	chromosomal region	-	16	-	13	MCM3, MCM5,MCM6, NCAPD2, KNTC1, BUB1, CENPF, ZWINT, HELLS, CDK1, CENPE, CENPK, CENPM
	GO:0000793	Condensed Chromosome	02	10	-	11	CENPE, NCAPD2, BUB1, ZWINT, CENPM, TOP2A, CENPF, CENPE, CENPK, KNTC1, SMC4
	GO:0005819	Spindle	04	8	07	-	TPX2, CENPF, NUSAP1, PRC1, TACC3, CDK1, CENPF

Table S2: Top Common reactome and wiki pathways from four databases.

Database	ID	Pathway	Associated Genes				Associated Common Genes
			DAVID	String	Enrichr	WebGestalt	
Reactome	R-HSA-69278	Cell Cycle, Mitotic	, 18	18	18	18	BUB1, TOP2A, PTTG1, TPX2, ZWINT, CENPE, CENPF, CENPK, CENPM, CDK1, KNTC1, MCM3, MCM5, MCM6, NCAPD2, RRM2, SMC4, TYMS

	R-HSA-114608	Platelet degranulation	11	11	11	11	ALB, AHSG, A2M, APOA1, APOH, FGA, FGB,FGG, HRG, PLG, SERPINA1
	R-HSA-1640170	Cell Cycle	18		18	18	BUB1, TOP2A, PTTG1, TPX2, ZWINT, CENPE, CENPF, CENPK, CENPM, CDK1, KNTC1,MCM3, MCM5, MCM6, NCAPD2, RRM2,SMC4, TYMS
	R-HSA-76002	Platelet activation, signaling and aggregation	12		11	12	ALB, AHSG, A2M, APOH, APOA1, F2, FGA, FGB,FGG,HRG, PLG, SERPINA1
	R-HSA-381426	Regulation of Insulin-like Growth Factor (IGF) transport and uptake by Insulin-like Growth Factor Binding Proteins (IGFBPs)	09	09		09	ALB, AHSG, APOA1, F2,FGA,FGG, PLG, SPERPINA1,SERPINC1
	R-HSA-68877	Mitotic Prometaphase	10	10	10	10	BUB1, ZWINT, CENPE,CENPF,CENPK, CENPM, CDK1, KNTC1, NCAPD2, SMC4,
	R-HSA-69620	Cell Cycle Checkpoints	11	11	11	11	BUB1,ZWINT,CENPE,CENPF, CENPK,CENPM,CDK1,KNTC1, MCM3,MCM5,MCM6
Wiki	WP4927	COVID 19 Thrombosis And Anticoagulation	05	05	05	-	F2,FGA,FGB,FGG,PLG
	WP5115	Network Map Of SARS CoV 2 Signaling Pathway	10	10	10	-	ALB,APOA1,APOH,CDK1,FGA, FGB,FGG,HP,HRG,RRM2
	WP176	Folate Metabolism	07	07	07	07	ALB,APOA1,F2,FGA,FGB,FGG,PLG
	WP272	Blood Clotting Cascade	05	05	05	05	F2,FGA,FGB,FGG,PLG
	WP2446	Retinoblastoma Gene in Cancer	06	06	06	06	TOP2A,CDK1,MCM3,MCM6,RRM2, TYMS
	WP179	Cell Cycle	06	06	06	06	BUB1,PTTG1,CDK1,MCM3,MCM5, MCM6
	WP15	Selenium Micronutrient Network	07	07	07	07	ALB,APOA1,F2,FGA,FGB,FGG,PLG
	WP4240	Regulation of sister chromatid separation at the metaphase-anaphase transition	03	03	03	03	BUB1,PTTG1,CENPE
	WP2806	Complement System	06	06	06	06	ALB,APOA1,FGA,FGB,FGG,PLG
	WP2361	Gastric Cancer Network 1	03	03	03	-	TOP2A,TPX2,CENPF

Table S3: Survival plot related values generated form GEPA, KM plotter and UALCAN databases for cluster two hub genes.

	GEPIA2						KM Plotter				UALCAN
Gene	OS			RFS			OS				Survival
	Logrank	HR	p(HR)	Logrank	HR	p(HR)	HR	Logrank	HR	Logrank	p-value

	p			p				p		p	
A2M	0.7400	0.94	0.74	0.6400	0.93	0.64	0.91	0.58	0.92	0.61	0.28
AHSG	0.1500	0.78	0.15	0.1700	0.81	0.17	0.70	0.042	0.64	0.0074	0.20
ALB	0.3200	0.84	0.32	0.6300	0.93	0.63	0.87	0.44	0.83	0.26	0.23
AMBP*	0.0750	0.73	0.076	0.8800	0.98	0.87	0.75	0.097	0.81	0.21	0.52
APOA1*	0.1800	0.79	0.17	0.6400	0.93	0.64	0.59	0.0031	0.86	0.37	0.035
APOC3**	0.0026	0.59	0.0031	0.0007	0.60	0.0008	0.59	0.0024	0.68	0.023	0.100
APOH*	0.074	0.73	0.075	0.1200	0.79	0.12	0.57	0.0016	0.71	0.043	0.12
F2	0.4300	0.87	0.44	0.082	0.77	0.084	0.63	0.0092	0.65	0.0095	0.71
FGA**	0.017	0.65	0.018	0.013	0.68	0.013	0.64	0.011	0.64	0.011	0.003
FGB**	0.029	0.68	0.031	0.0011	0.6	0.0011	0.58	0.0021	0.72	0.049	0.038
FGG**	0.048	0.70	0.049	0.02	0.70	0.019	0.59	0.0029	0.69	0.025	0.037
GAPDH**	2.1e-05	2.2	3.3e-05	0.067	1.30	0.067	1.7	0.0029	1.34	0.078	0.0037
HP	0.30	0.83	0.30	0.12	0.79	0.12	0.76	0.11	0.73	0.064	0.89
HPX**	0.0043	0.60	0.0047	0.041	0.73	0.04	0.61	0.0055	0.68	0.02	0.016
HRG**	0.0083	0.63	0.0091	0.0037	0.64	0.0041	0.47	2.2e-05	0.64	0.0081	0.0032
PLG**	0.076	0.73	0.077	0.022	0.71	0.023	0.67	0.023	0.68	0.023	0.093
SERPINA1	0.25	1.2	0.25	0.62	0.93	0.62	1.06	0.74	0.85	0.33	0.84
SERPINC1**	0.054	0.71	0.056	0.015	0.69	0.016	0.62	0.0074	0.71	0.038	0.023
TFRC**	0.037	1.4	0.039	0.083	1.30	0.083	1.36	0.084	1.06	0.71	0.00076
TTR**	0.052	0.71	0.054	0.014	0.69	0.015	0.69	0.037	0.65	0.0097	0.083

Table S4: Hub genes identification for different intersection of datasets for two and six methods using String and Cytoscape.

scDEA∩scHD4E				Seurat∩Limma∩TPMM∩ROSeq∩scDEA∩scHD4E			
A∩B∩C	A∩B∩D	B∩D∩C	A∩D∩C	A∩B∩C	A∩B∩D	B∩D∩C	A∩D∩C
TOP2A	TOP2A	MCM6	APOH	TOP2A	TOP2A	MCM6	APOH
MCM6	BUB1	CDK1	AHSG	CENPF	TPX2	TOP2A	AHSG
CENPF	CENPF	TOP2A	APOA1	TPX2	CENPF	CDK1	ALB
ATAD2	CENPE	TYMS	TTR	PRC1	NCAPD2	TYMS	SERPINA1
NUSAP1	TPX2	RRM2	SERPINC1	CKAP5	PRC1	RRM2	APOA1
TPX2	NCAPD2	SMC4	AMBP	NUSAP1	CENPK	SMC4	TTR
PRC1	MKI67	CENPF	A2M	HDAC1	BRCA2	CENPF	HPX
CKAP5	PRC1	KNTC1	ALB	ACAA1	TP53BP1	KNTC1	HP
MCM3	MCM6	ATAD2	FGA	TP53BP1	ALB	ATAD2	FGA
HELLS	PTTG1	ZWINT	FGB	HADHB	TFRC	ZWINT	FGG
GAPDH	ATAD2	EZH2	SERPINA1	ALB	RASSF3	EZH2	F2
HDAC1	HELLS	MCM5	HPX	ZBTB16	MT1E	MCM5	FGB
RAD21	CENPK	PRC1	FGG	SCD	PSAT1	PRC1	APOC3
PAICS	TACC3	MCM3	F2	IDH2	SYNJ2	MCM3	HRG
MTHFD2	CENPM	TPX2	HP	TFRC	PGM1	TPX2	PLG

Table S5: hHub Genes for cluster one and cluster two.

Cluster One	TOP2A	CDK1	BUB1	CENPF	NUSAP1	KNTC1	RRM2	SMC4	ZWINT
	TYMS	MCM6	MKI67	MCM5	CENPE	TPX2	PRC1	ATAD2	NCAPD2
	EZH2	PTTG1	HELLS	MCM3	CENPK	TACC3	CENPM		
Cluster	ALB	APOA1	SERPINA1	HP	FGB	APOC3	FGA		

Two	TTR	A2M	PLG	AHSG	F2	FGG	HRG
	SERPINC1	APOH	AMBP	TFRC	GAPDH	HPX	

Table S6: Survival plot related values generated form GEPA, KM plotter and UALCAN databases for cluster one hub genes.

	GEPIA2						KM Plotter				UALCAN
Gene	OS			RFS			OS				Survival
	Logrank p	HR	p(HR)	Logrank p	HR	p(HR)	HR	Logrank p	HR	Logrank p	p-value
BUB1	0.001	1.8	0.001	0.002	1.6	0.002	1.85	0.001	1.57	0.007	0.0001
CDK1	0.000	2.0	0.000	0.001	1.7	0.001	1.69	0.003	1.59	0.006	<0.0001
CENPE	0.011	1.6	0.012	0.008	1.5	0.008	1.76	0.001	1.52	0.013	0.0022
CENPF	0.002	1.7	0.002	0.002	1.6	0.002	1.54	0.013	1.66	0.003	0.0170
CENPK	0.001	1.8	0.001	1.4e-05	2.0	1.8e-05	1.77	0.001	1.88	0.000	0.0032
CENPM	0.001	1.9	0.001	0.003	1.6	0.004	1.87	0.000	1.73	0.001	0.0003
EZH2	5.6e-05	2.1	7.8e-05	1e-04	1.8	0.000	1.97	0.000	1.79	0.001	<0.0001
HELLS	0.018	1.5	0.019	7e-04	1.7	0.001	1.49	0.022	1.53	0.011	0.0056
KNTC1	0.007	1.6	0.008	0.001	1.6	0.002	1.7	0.003	1.74	0.001	0.0007
MCM3	0.000	1.9	0.000	5.9e-05	1.9	6.9e-05	1.49	0.023	1.58	0.006	0.0340
MCM5	0.048	1.4	0.049	0.032	1.4	0.032	1.34	0.098	1.32	0.099	0.0003
MCM6	0.004	1.7	0.005	2e-05	1.9	2.6e-05	1.82	0.001	2.01	3.3e-05	0.0002
MKI67	0.001	1.9	0.001	4.2e-05	1.9	5.1e-05	1.81	0.001	1.73	0.001	0.0035
NCAPD2	0.046	1.4	0.047	0.013	1.5	0.013	1.29	0.150	1.19	0.300	<0.0001
NUSAP1	0.006	1.6	0.007	7e-04	1.7	0.001	1.55	0.012	1.47	0.021	0.073
PRC1	0.000	1.9	0.001	0.001	1.7	0.001	1.79	0.001	1.6	0.005	0.0670
PTTG1	3.5e-06	2.3	6.4e-06	2.3e-05	1.9	3.1e-05	1.76	0.002	1.81	0.001	<0.0001
RRM2	0.001	1.9	0.001	0.000	1.7	0.000	1.66	0.004	1.39	0.047	0.0002
SMC4	0.002	1.7	0.003	0.089	1.3	0.088	1.7	0.003	1.39	0.049	0.0002
TACC3	0.003	1.7	0.003	0.000	1.8	0.000	1.65	0.005	1.51	0.013	0.0004
TOP2A	0.003	1.7	0.003	0.001	1.7	0.001	1.82	0.001	1.66	0.003	0.0003
TPX2	0.001	1.9	0.001	0.003	1.6	0.003	2.05	5.7e-05	1.71	0.002	<0.0001
TYMS	9e-04	1.8	0.001	0.000	1.8	0.000	1.48	0.027	1.42	0.037	0.0002
ZWINT	0.001	1.8	0.001	7.8e-05	1.8	9.4e-05	1.80	0.001	1.80	0.001	<0.0001
ATAD2	0.23	1.2	0.23	0.061	1.3	0.061	1.2	0.310	1.34	0.084	0.2600
